# T Cell and Natural Killer Cell Membrane-Camouflaged Nanoparticles for Cancer and Viral Therapies

**DOI:** 10.1021/acsabm.4c00074

**Published:** 2024-04-30

**Authors:** Fatma Ozsoy, Mahir Mohammed, Nasrullah Jan, Elif Lulek, Yavuz Nuri Ertas

**Affiliations:** † ERNAM−Nanotechnology Research and Application Center, Erciyes University, Kayseri 38039, Turkey; ‡ Department of Biomedical Engineering, Erciyes University, Kayseri 38039, Turkey; § Department of Pharmacy, The University of Chenab, Gujrat, Punjab 50700, Pakistan; ∥ UNAM−National Nanotechnology Research Center, Bilkent University, Ankara 06800, Turkey

**Keywords:** Cancer therapy, T cells, Natural killer cells, Biomimetic nanoparticles, Cell membrane coating

## Abstract

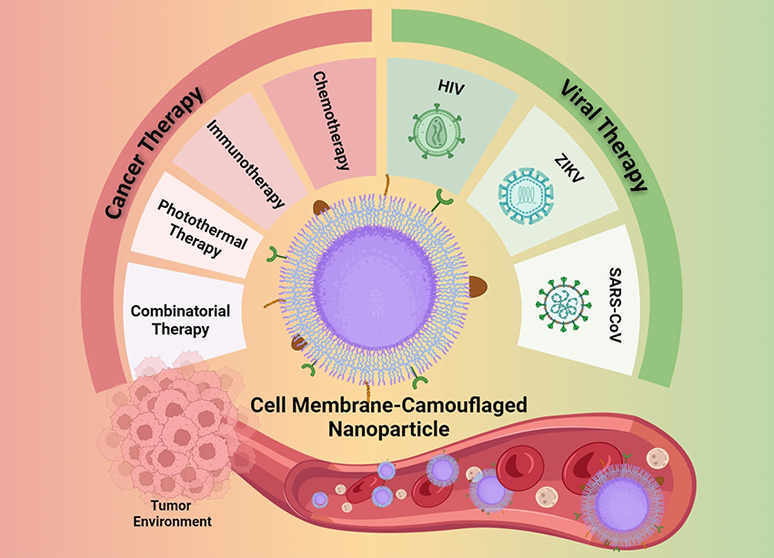

Extensive research has been conducted on the application of nanoparticles in the treatment of cancer and infectious diseases. Due to their exceptional characteristics and flexible structure, they are classified as highly efficient drug delivery systems, ensuring both safety and targeted delivery. Nevertheless, nanoparticles still encounter obstacles, such as biological instability, absence of selectivity, recognition as unfamiliar elements, and quick elimination, which restrict their remedial capacity. To surmount these drawbacks, biomimetic nanotechnology has been developed that utilizes T cell and natural killer (NK) cell membrane-encased nanoparticles as sophisticated methods of administering drugs. These nanoparticles can extend the duration of drug circulation and avoid immune system clearance. During the membrane extraction and coating procedure, the surface proteins of immunological cells are transferred to the biomimetic nanoparticles. Such proteins present on the surface of cells confer several benefits to nanoparticles, including prolonged circulation, enhanced targeting, controlled release, specific cellular contact, and reduced in vivo toxicity. This review focuses on biomimetic nanosystems that are derived from the membranes of T cells and NK cells and their comprehensive extraction procedure, manufacture, and applications in cancer treatment and viral infections. Furthermore, potential applications, prospects, and existing challenges in their medical implementation are highlighted.

## 1 Introduction

Cancer is a disease caused by uncontrolled cell proliferation that is invasive and metastatic in nature. Cancerous cells differ from normal/healthy cells in terms of enhanced invasive nature and decreased drug sensitivity to the site of action. Until now, cancer therapy has been carried out on the basis of pathological and clinical staging utilizing different diagnostic methods, such as conventional histopathological and radiological examinations. Conventional cancer therapy is restricted to radiation, surgery, and chemotherapy.^1−5^

Caused by bacterial, viral, fungal, and other microorganisms, infectious diseases are a major global health issue. Despite progress, molecular mechanisms of host–microbe interactions remain elusive, which leads to poor treatment responses and drug resistance.^6,7^ RNA virus infections, such as HIV, Zika, and SARS-CoV-2, pose significant health threats. Currently, antiretroviral therapy and neutralizing antibodies are used, but treatment outcomes remain unsatisfactory. Innovative therapeutics against virus infectious diseases are needed before valid vaccination or treatment.^8,9^

Nanoparticles (NPs) have been broadly explored in cancer diagnosis and treatment.^10,11^ Notwithstanding their benefits, the realization of successful clinical translation of nanomaterials remains elusive. The major hurdles that account for the discrepancy between academic and clinical results are due to the reticuloendothelial system (RES), which identifies NPs as unfamiliar substances and eliminates them.^12^ Furthermore, the intricate vascular milieu, including immune cells and elevated concentrations of proteins, hastens the elimination of NPs.^13^ PEGylation, which involves modifying the surface of NPs with polyethylene glycol, has been a crucial technique for prolonging the circulation time of NPs for many years. Nevertheless, recent research has demonstrated the emergence of antibodies against PEG, thereby prompting drug delivery professionals to search for an alternative biomimetic approach to evade immune recognition.^14,15^ As a result, researchers have explored a new and promising technique to disguise NPs by enveloping them with plasma membranes derived from living cells. This represents an innovative technique creating versatile NPs that can interact effectively with living organisms while evading detection and elimination as alien entities by the RES and mononuclear phagocytic system (MPS).^16^

These NPs are enveloped with a cell membrane and have a core–shell configuration that blends the benefits of the inherent properties of the biological cellular membrane and the physical and chemical features of synthetic NPs.^17^ Through this method, T cell membrane-camouflaged NPs carrying effective drugs can achieve better targeting of tumors through self-recognition, homotype targeting, and long-term presence in the systemic circulation of the body, which leads to better results in cancer treatment. These bioinspired NPs are adept at tricking the host’s defense mechanisms and extending circulation time. Additionally, they can selectively transport therapeutic agents to the desired location.^18^ The resultant nanostructure is composed of NPs containing the therapeutic agent at its core and a cellular membrane coating sourced from diverse cell types.^19^ These cell membranes can be obtained from different types of cells, including red blood cells (RBCs),^20^ white blood cells (WBCs),^21^ platelets,^22^ stem cells,^23^, and cancer cells.^24^ RBCs are the predominant type of blood cell in humans and are crucial for carrying oxygen from the lungs to distant locations through the hemoglobin protein found in each cell. RBCs can be readily obtained from donor blood and are considered an excellent source of cellular membranes for circulating in the vasculature of patients. However, surface alteration of red blood cells can lead to hemolysis.^25^ Platelets are derived from megakaryocyte progenitor cells. They are essential blood components involved in several processes, such as immunity, wound healing, and tumor metastasis. Platelet membranes have the potential to be advantageous for coating NPs because they can help in evading the immune system through CD47-mediated macrophage evasion and CD55/59-mediated prevention of complement activation. However, their use can lead to unwanted activation of platelet membranes.^26,27^ Mesenchymal stem cells (MSCs) are easily obtainable and capable of long-term in vitro growth. MSCs have long-term circulatory potential, immune evasion capabilities, and tumor targeting qualities, thereby making them well-suited for delivering nanoparticles. The cells express a variety of ligands suitable for targeting tumors and easily move to inflamed regions in vivo because of this characteristic.^28^ Nevertheless, they are constrained by low specificity. Cancer cells can serve as a promising supply of membrane material for coating nanoparticles. They are of interest since many cancer cells can efficiently adhere to other cancer cells through homologous adhesion. However, cancer cell membranes have a shorter circulation time.^16,29^ WBCs have a significant impact on the host’s immunity and are favored because of their different roles in the immune system, which perform their individual functions against viruses, bacteria, and tumor cells. WBCs exist in different types, including lymphocytes, monocytes, eosinophils, basophils, and neutrophils, each with its unique morphological and physiological characteristics.^30^

Natural killer (NK) cells are large granular lymphocytes in the innate immune system that provide host defense against microbial infections and tumor cells. They contribute about 5–20% to peripheral blood mononuclear cells and can target cancer cells directly via receptors on their cell surface.^31^ The NK cell detection system includes various cell surface-activating and inhibitory receptors, which regulate NK cell activities. NK cells express several toll-like receptors (TLRs), which induce IFN-γ production and enhance cytotoxicity. They also express the low-affinity Fc receptor CD16, which enables them to detect antibody-coated target cells and exert antibody-dependent cell cytotoxicity.^32,33^

This review presents an overview of the characteristics and roles of T cells. It also delves into the concept of membrane coating for cell-specific targeting, which covers aspects such as the various kinds of nanomaterials, methods for synthesizing membranes, core particles, and their applications in medicine. The concepts of cell membrane coating technologies and their respective preparation methods are then outlined. These processes involve the extraction of T cell membranes and their encapsulation in nanoscale substances. Then, the biological applications of NPs in cancer therapy are discussed. Ultimately, the challenges and limitations of translating research to clinical settings are examined. Our aim is to undertake a thorough examination of NPs coated with T cell and NK cell membranes for targeted cancer therapies.

## 2 T Cell: Biology and Functions

The generation and upkeep of immune responses, as well as memory and homeostasis, are reliant on T cells. T cells possess a receptor that can recognize antigens from various sources, such as bacteria, malignancies, and the surroundings, while also retaining immunological memory. T cells may also have a part to play in certain autoimmune and inflammatory ailments. The operative function of T cells in the context of immunity and immunopathology has been primarily investigated through murine models, which has aided in the creation of immune-based treatments for humans.^34^

Progenitor cells from the bone marrow give rise to T lymphocytes, which undergo maturation in the thymus before migrating to peripheral organs. Among the subtypes of peripheral T cells are those that are naive and those that are memory cells capable of responding to various antigens. When dendritic cells come across an antigen and costimulatory molecules, inexperienced T cells generate cytokines that move to various locations to aid in the removal of pathogens by generating cytokines and toxins. Effector cells that are activated may survive and become memory T cells, which can be further classified according to their mobility, distribution across tissues, and ability to self-renew. Memory subsets are involved in sustaining long-term immunity and recalling protective responses, but their lineage and origin remain unclear.^35^

The function of T cells in the immune system varies across the organism. T cells are present in lymphoid tissue, exocrine tissue, mucosal and barricade sites, fatty tissue, and the central nervous system. Most T lymphocytes are situated in lymphoid tissues, including the spleen, tonsils, and bone marrow. Despite only constituting around 2%–3% of the complete T cell component, peripheral blood, high concentrations of T cells can also be found in barrier areas like the skin, colon, and lungs.^36^ T lymphocytes are responsible for eliminating developing tumors and intracellular pathogens, such as specific viruses and bacteria. Furthermore, they manage the potency of adaptive immune reactions, and their production of cytokines can be utilized to distinguish them.^37^

**Figure 1:**
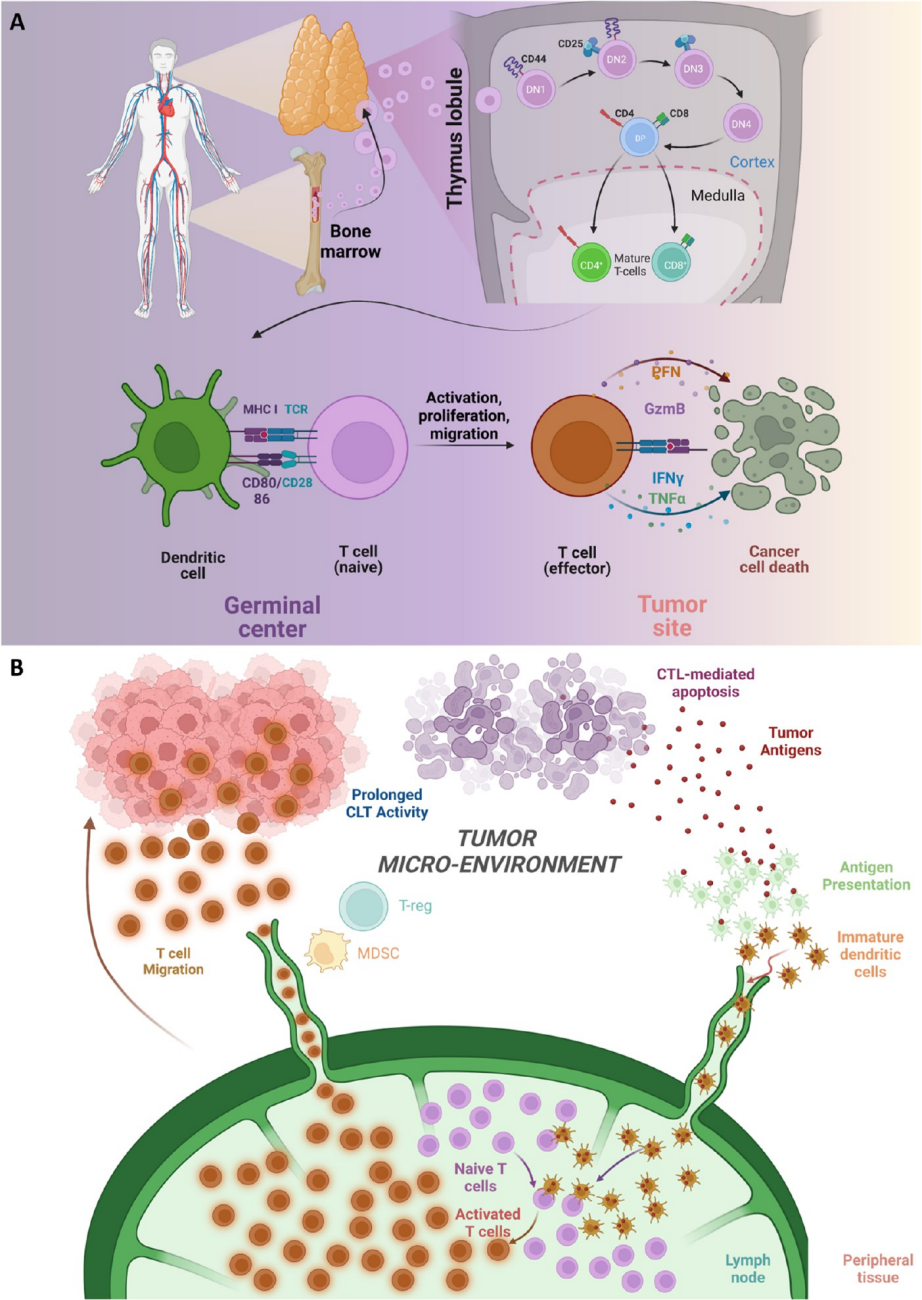
A general pattern of the production and interactions of T cells. (A) Maturation process of T cells from lymphoid precursors that relocate from the bone marrow to the thymus. (B) Inactive T cells travel through secondary lymphoid organs, such as lymph nodes, spleen, and tonsils, multiple times until they detect MHC–peptide complexes on the surface of cells that protect against antigens and become fully stimulated. The authors generated this figure with BioRender (https://www.biorender.com/).

The categorization of lymphocytes, referred to as cluster of differentiation (CD), is established on distinct surface markers, which encompasses more than 300 variations and also comprises T cell antigen receptor (TCR).^38^ The production of T cells originates from precursor cells that exhibit biomarkers and possess the ability to infiltrate the thymic cortex. The reorganization of α, β, γ, and δ chains results in the creation of T lymphocytes with αβ-chains (T-αβ) and T lymphocytes with γδ-chains (T-γδ). CD3 and the αβ-TCR or γδ-TCR are used to distinguish between T-αβ and T-γδ cells, respectively. NK-T cells are derived from precursor T cells that express CD3. They do this by creating a specific α-chain that interacts with glycolipid-CD1d via a β-chain interaction. After maturation, NK-T cells acquire CD56 expression. However, precursor T cells have limited CD3 on their surface and must express CD8 and CD4 before CD3 in a rapid transition. T-γδ cells are separated from the αβ-path with around 30% of T-γδ cells being CD8^+^. The CD4^+^CD8^+^ double-positive T-αβ lymphocytes undergo positive selection by interacting with peptide–major histocompatibility complex (MHC) I or peptide–MHC II complexes, which results in the generation of CD8 or CD4, respectively. T-αβ cells migrate from the thymus cortex to the medulla where they experience negative clonal selection to eliminate T cells with strong attraction for self-antigens. In due course, fully developed CD4^+^ and CD8^+^ T cells with a single-positive feature are discharged into the bloodstream (Figure 1A).^39^

After adhering to antigen, T lymphocytes multiply and generate several kinds of T cells, including cytotoxic T cells and memory T cells. Through the production of a specialized protein called perforin, killer T cells trigger the death of virus-infected and cancerous cells by creating pores within them. There exist multiple subcategories of T cells, each with a unique yet interconnected role. They are made up of diverse cell types, such as T-helper and cytotoxic T cells, as well as regulatory T cells, and each performs a distinct function in comparison with other lymphocytes. T lymphocytes regulate the immune system and have a significant impact on cancer and autoimmune and infectious diseases (Figure 1B).

## 3 Fabrication of T Cell Membrane-Coated Nanoparticles

The breakdown or disruption of cells is a crucial process in obtaining T cell membrane vesicles from them. The lysate is created by rupturing the cell membrane. Chemical and photonic cell rupture methodologies (such as chemical rupture and diffusion) and more rigorous procedures (such as compression, mechanical and ultrasonic blending, and mortar and pestle) are the two fundamental classifications of cell rupture techniques.^40^ Chemical lysis utilizes buffers, salts, detergents, and enzymes and does not entail crushing or scraping. Before finalizing a method for cell lysis, there are several aspects that need to be considered. The primary factor is the type of cell. Precisely, the investigation proposes the employment of sonication, extrusion, hypotonic treatment, and microfluidic electroporation to disintegrate erythrocytes. By employing sonication or multiple freeze–thaw methods, it has been possible to isolate membrane vesicles from platelet cells. The identification and characteristics of crucial proteins, which can be either peripheral or integral, constitute the second aspect. Most extraction methods attempt to provide a cell membrane with fully functional proteins. Mechanical lysis is often indicated for large cell clumps or tissue pieces. The mechanical homogenization procedure entails freezing the tissues followed by crushing them using a mortar and pestle to exert physical pressure and generate lysate.^41^ After the T cell membrane has been extracted, the subsequent process comprises enveloping the NPs with the membrane vesicle of the cell. The objective is to improve the biointeraction capabilities of NPs. In recent years, numerous coating processes have been presented. Among these, there are three commonly used methods: membrane extrusion, ultrasonic fusion, and electroporation. One of the most frequently used procedures involves the physical extrusion of the pure membrane and NP cores via a permeable membrane. Extrusion applies a mechanical force that culminates in the amalgamation of particles and vesicles. The extrusion is succeeded by a centrifugal procedure to segregate the unencapsulated vesicles from the precipitation. Extrusion produces consistent coatings and promotes the creation of nanoparticles with even sizes. This approach is highly successful for small-scale production and is the most commonly chosen method because of its advantages.^42^ Employing sonication-dependent techniques that utilize ultrasonic-induced disruptive energy to produce a core–shell nanomaterial from two constituents is yet another option. Improper management of sonication can lead to denaturation of membrane proteins and drug leakage, despite its speed and simplicity. Optimal outcomes need the optimization of sonication time, frequency, and power. However, this approach does not always guarantee the consistent diameter of the resulting cell membrane-coated nanoparticles (CMCNs).^43^ Furthermore, a microfluidic mechanism and on-site application of NP coating have been suggested.^44^ The electroporation method is a microfluidics-based method used for the production of membrane-covered NPs. In recent years, this method has been used successfully, and efficient and reliable results were reported with appropriate optimization. Because this method is new, the device needs to be developed by researchers, which is not commercially available. Therefore, the scalability of this method needs to be investigated in the future.^13^

**Figure 2:**
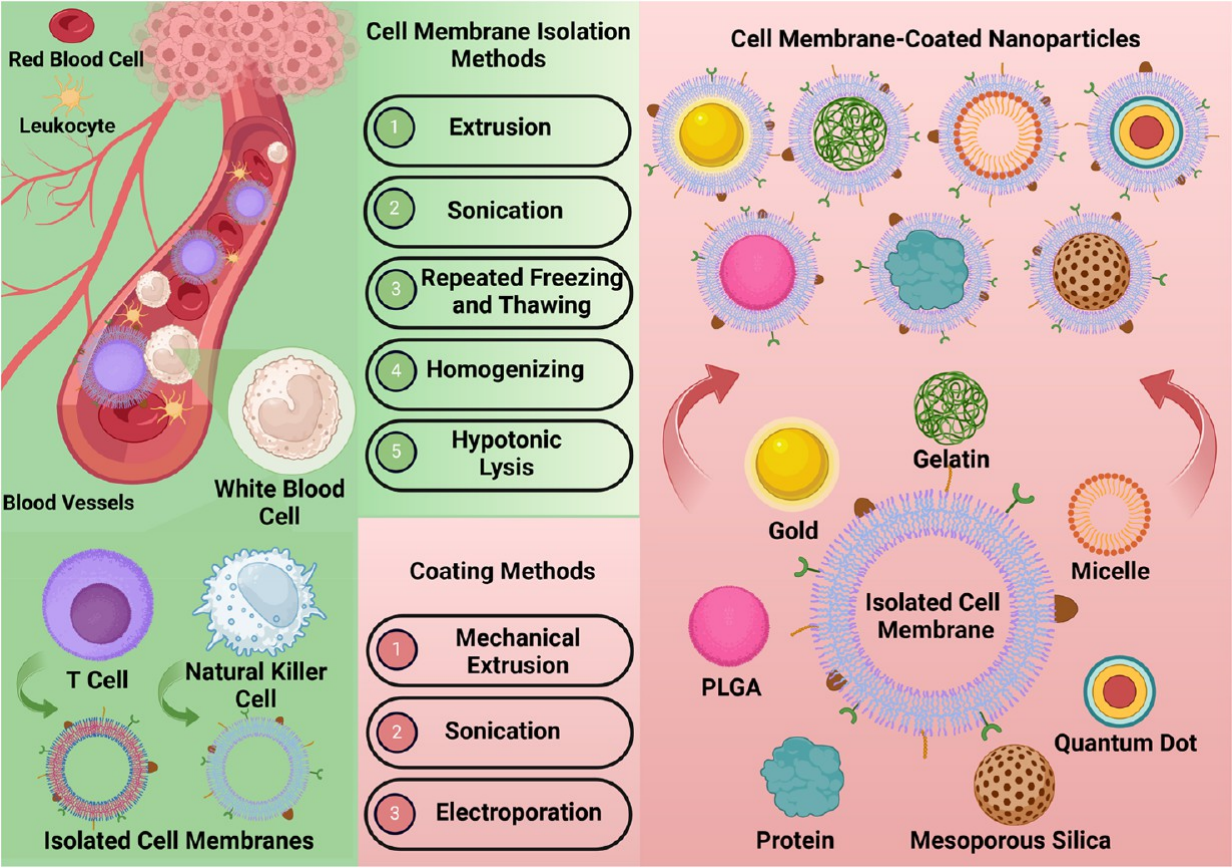
T cell and natural killer cell for cell membrane coating, cell membrane isolation methods, coating methods, production of the NP core, and encapsulation with the cellular membrane for cell membrane coating. The authors generated this figure with BioRender (https://www.biorender.com/).

Regarding the selection of the NP as core, the most commonly employed nanomaterials are polylactic-*co*-glycolic acid (PLGA), gelatin, mesoporous silica, and micelles.^45^ PLGA has been frequently exploited as the core material in cell membrane coating because of its (i) biodegradability and biocompatibility, (ii) recognition by both the United States Food and Drug Administration (FDA) and European Medicines Agency (EMA) for usage, (iii) adaptability to accommodate a variety of water-soluble and insoluble drugs, and (iv) manageable biodegradation features that allow for customized and prolonged drug delivery.^46^ Gelatin, a natural polypeptide, is a versatile drug/vaccine delivery carrier because of its easy availability with low cost, biodegradability, nonimmunogenicity, and biocompatible nature. The surface of gelatin NPs can be easily altered using particular ligands or covered with cell membrane for targeted delivery. Gelatin NPs offer superior stability in biological fluids compared with other colloidal carriers, which ensures the desired sustained and controlled release of cargo.^47^ Mesoporous silica NPs (MSNPs) have garnered significant attention in drug delivery and biomedicine. The benefits of MSNPs include improved regulation of drug loading and release kinetics because of their mesoporous structure and customizable pore size, as well as convenient surface customization for precise and controlled drug administration. Additionally, MSNPs have received satisfactory safety evaluations in vivo.^48^ As a result, MSNPs are an attractive choice for cell membrane-coated technology because of their excellent surface properties and porosity. Polymeric micelles are nanoscopic structures that form by the self-assembly of amphiphilic copolymers when the concentration of the micelles reaches a critical point. Their versatility in tailoring different molecular features makes them ideal for encapsulating mostly water-insoluble drugs.^49^ Their nanosize, good solubility properties, and ease of preparation make polymeric micelles a promising carrier for different administration routes. Polymer-based micelles can increase drug availability within the body and offer a regulated and directed drug discharge, which is useful in reducing side effects. Figure 2 illustrates the process of fabricating T cell membrane-coated NPs.

## 4 Cancer Therapy

NPs enclosed by the cell membrane have surfaced as a hopeful alternative for directing tumors as they maintain the complex and innate traits of the donor cells.^50^ Compared with other cells, T cells have distinct targeting traits^51^ that can be utilized to specifically target cancerous growths.^52^ T cells that have been activated possess a high attraction toward cancerous cells because of the existence of distinct immune recognition molecules, such as TCRs, on their surfaces. Such activated T cells are capable of detecting chemical linkages present on the surface of tumors.^53^ As a result, T cell membranes can be used for nanodrug delivery to tumors by utilizing the immune-recognition properties of T cells.^54^ In this section, research on cancer treatment through biomimetic T cell membrane-coated NPs is examined using different subheadings, which are classified into four groups according to the approach of cancer therapy: (i) chemotherapy, (ii) immunotherapy, (iii) photothermal therapy, and (iv) combinatorial therapy.

### 4.1 Chemotherapy

Although there have been advancements in the progress of cancer drugs, it is evident that cytotoxic chemotherapy will remain the fundamental approach to treating cancer.^54,55^ Nevertheless, the current chemotherapies are associated with nonselective drug delivery, which leads to adverse effects and toxicity that limit their efficacy.^56,57^ This calls for a fresh approach to treatment and the development of distinct therapeutic methodologies. Innovative biomimetic NPs with multiple functions have been created to improve the effectiveness of chemotherapy drugs and overcome the restrictions.^58,59^ These versatile nanocarriers possess the ability to transform the treatment of diverse types of cancer by utilizing the strategy of concealing themselves in T cell membrane. However, there are very few studies in this field of chemotherapy.^60^

**Figure 3:**
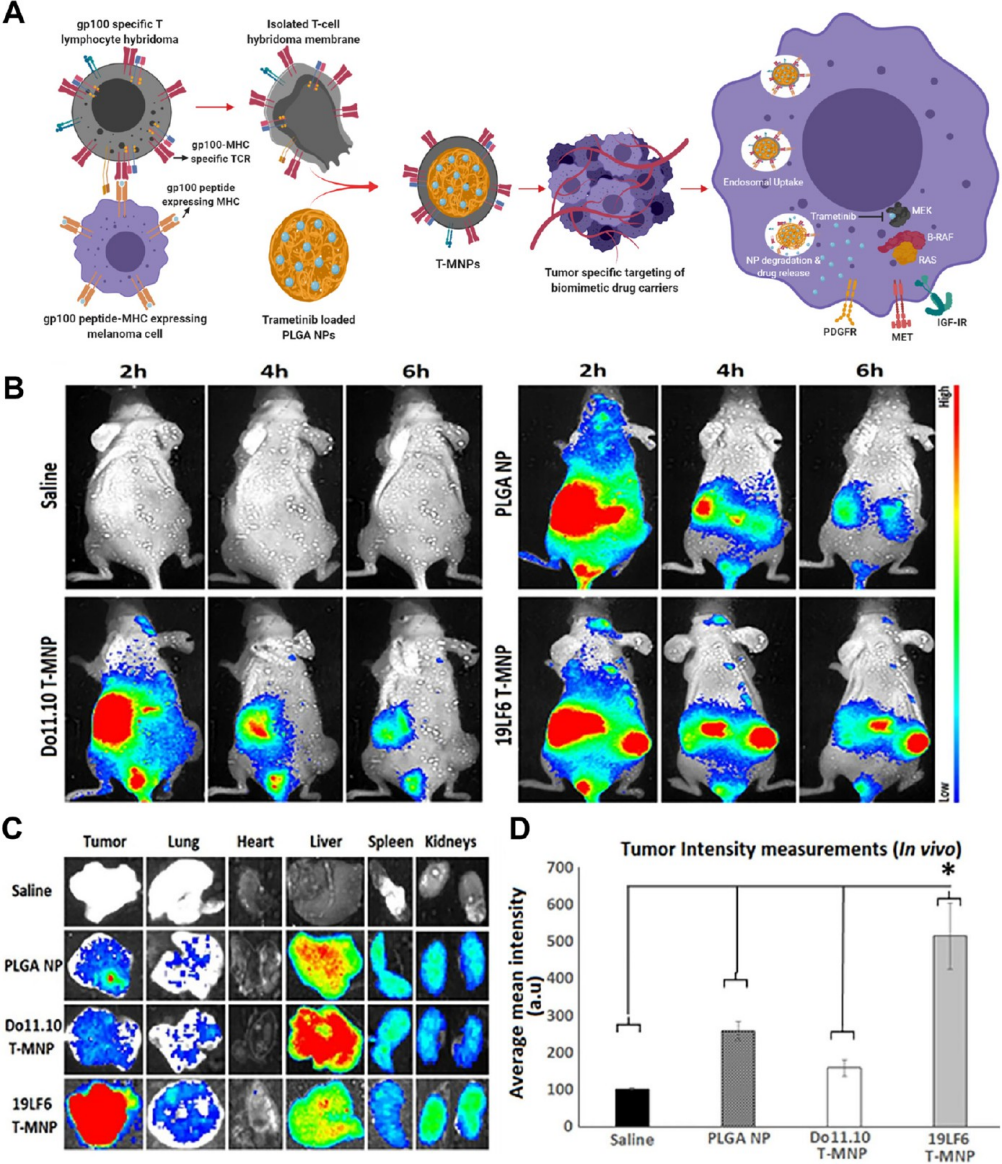
Schematics of nanoparticles coated with T cell membranes in melanoma treatment. (A) Schematics of T-MNP. (B) Nanoparticles’ ability to target tumors in real time when administered intravenously. (C) Ex vivo images displaying the biodistribution in organs. (D) In vivo intensity of fluorescence in tissue homogenates during biodistribution. Reproduced with permission under CC-BY license from ref (60). Copyright 2020.

Present therapies for melanoma, like surgical interventions, chemotherapy, immunotherapy, and radiation therapy, all come with disadvantages, such as low rates of response, increased toxicity, severe side effects resulting from nonspecific drug targeting, and the gradual emergence of resistance to multiple medications. In order to offer a treatment option for melanoma, three types of NPs were produced, each coated with a different type of membrane. Three kinds of membrane-coated NPs (MNPs) were developed: (1) T-MNPs (melanoma-specific T-cells) coated on the 19LF6 cell line (treatment); (2) D-MNPs (nonspecific T-cells) coated on the DO11.10 cell line (control for T-cells); and (3) A549 cell line (lung cancer) coated on A-MNPs (control for other cell types).The first type was coated with a membrane from the 19LF6 cell line, which consisted of T-cells specific to melanoma, and these NPs were referred to as T-MNPs. The second type of NPs, referred to as D-MNPs, were coated with a membrane from the DO11.10 cell line, which are nonspecific T-cells. These NPs served as a control for T cells that are specific to melanoma. The third type of NPs, referred to as A-MNPs, were coated with a membrane from the A549 cell line associated with lung cancer, which served as the control for other cell types. To enhance the effectiveness of treatment, a versatile NP was proposed for targeted and efficient treatment of melanoma. It consisted of chemotherapeutic agent trametinib-loaded PLGA NPs coated with a biological membrane that expressed a melanoma-specific anti-gp100/HLA-A2 TCR (19LF6) (Figure 3). The stability, hemocompatibility, and cytocompatibility of the NPs were outstanding. Furthermore, it was demonstrated that the discharge patterns of medications from NPs are managed by the coating of the membrane, with the most significant sustained release being proportional to the quantity of the membrane employed. In comparison with uncoated NPs, membrane-coated 19LF6 NPs resulted in a 3-fold increase in the intake of melanoma cell lines in vitro. In addition, it was discovered that the kinetics of binding and cellular absorption were influenced by the concentrations of the membrane/TCR. These NPs were significantly more effective than other uncoated groups at killing cancer cells in vitro, and their binding and uptake properties were identical. In comparison with free medicines and negative controls, particles with a greater proportion of membranes are more effective. Through in vivo biodistribution experiments, it was demonstrated that these NPs possessed theragnostic qualities, exhibiting over a 2-fold increase in tumor retention compared to other groups. Therefore, the application of NPs coated with T cell membrane could act as a feasible potential theragnostic approach for the diagnosis and therapy of melanoma.^60^

### 4.2 Immunotherapy

The objective of cancer immunotherapy is to revive the capability of the immune system to recognize and refuse cancerous cells.^61^ This therapy is seen as a promising new chapter in the field of treatment because it can selectively eliminate cancer cells with less harmful effects than traditional treatment methods. Although cancer immunotherapy has shown promising results in treating tumors, it faces several challenges primarily related to the variability of tumors, malfunctioning immune cells, resistance to immunotherapy, and the potential for immune system damage. Therefore, it is essential to improve the efficiency of cancer immunotherapy.^62^

**Figure 4:**
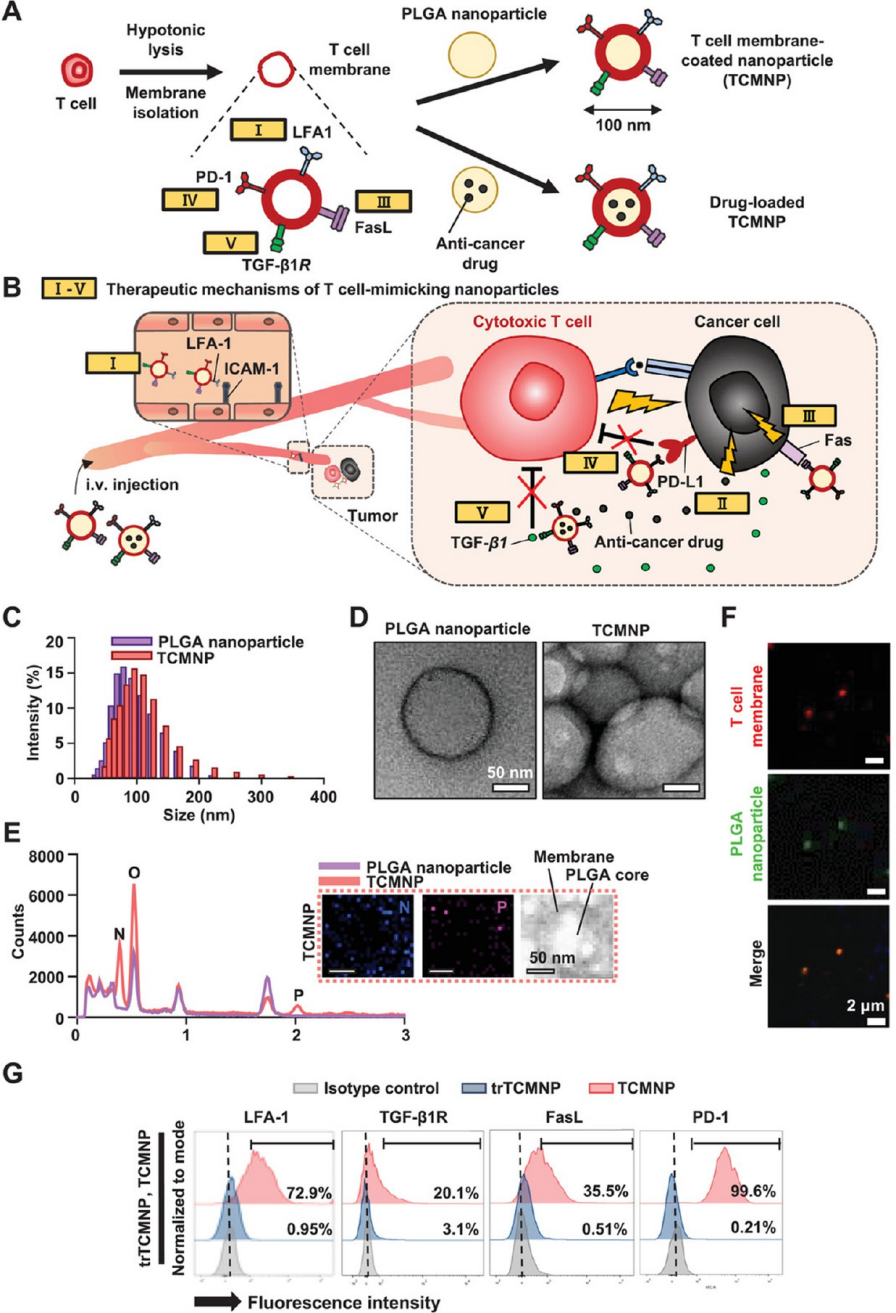
Generation and proposed medicinal operation of TCMNPs. (A) Fabrication of NPs. (B) Therapeutic mechanism of the T cell membrane-coated NPs. (C) Size evaluation. (D) TEM inspection. (E) Analysis of energy-dispersive spectroscopy of nanoparticles. (F) Laser microscopy through confocal technology. (G) Assessment through flow cytometry. Reproduced from ref (63). Copyright 2020 Wiley.

The most recent advancement in cancer immunotherapy has concentrated on the development of nanomaterials that employ T cell membranes.^63^ T cell membrane-coated NPs (TCMNPs) were formulated as an approach to immunotherapy aiming to surmount the constraints of existing cancer therapies (Figure 4A). TCMNPs target tumors by utilizing T cell membrane-derived proteins to release antitumor agents and activate Fas-ligand-mediated destruction to eliminate cancer cells. TCMNPs were shown to be more effective in treating melanoma than immune checkpoint inhibition. Compared to uncoated PLGA NPs, the hydrodynamic size distribution of TCMNPs rose slightly, while their zeta potential exhibited a decline (Figure 4B). TEM analysis revealed that TCMNPs had a spherical core–shell structure (Figure 4C). Components of plasma membranes, nitrogen (N), and phosphorus (P) were identified in the TCMNPs’ membrane (Figure 4D). Upon coating with NPs, fluorescent confocal images indicated the presence of both plasma membrane and PLGA NPs in TCMNPs, and the colocalization persisted even after treating B16F10 cancer cells (Figure 4E). On TCMNPs, membrane proteins were effectively preserved, whereas those on trypsin-pretreated TCMNPs (trTCMNPs) were diminished (Figure 4F). It was demonstrated that TCMNPs had antitumor properties when used for lung cancer treatment. Additionally, when TCMNPs were combined with T cell membrane proteins, they acted as NPs that camouflage T cells, thereby amplifying the effectiveness of cancer immunotherapy.^63^

**Figure 5:**
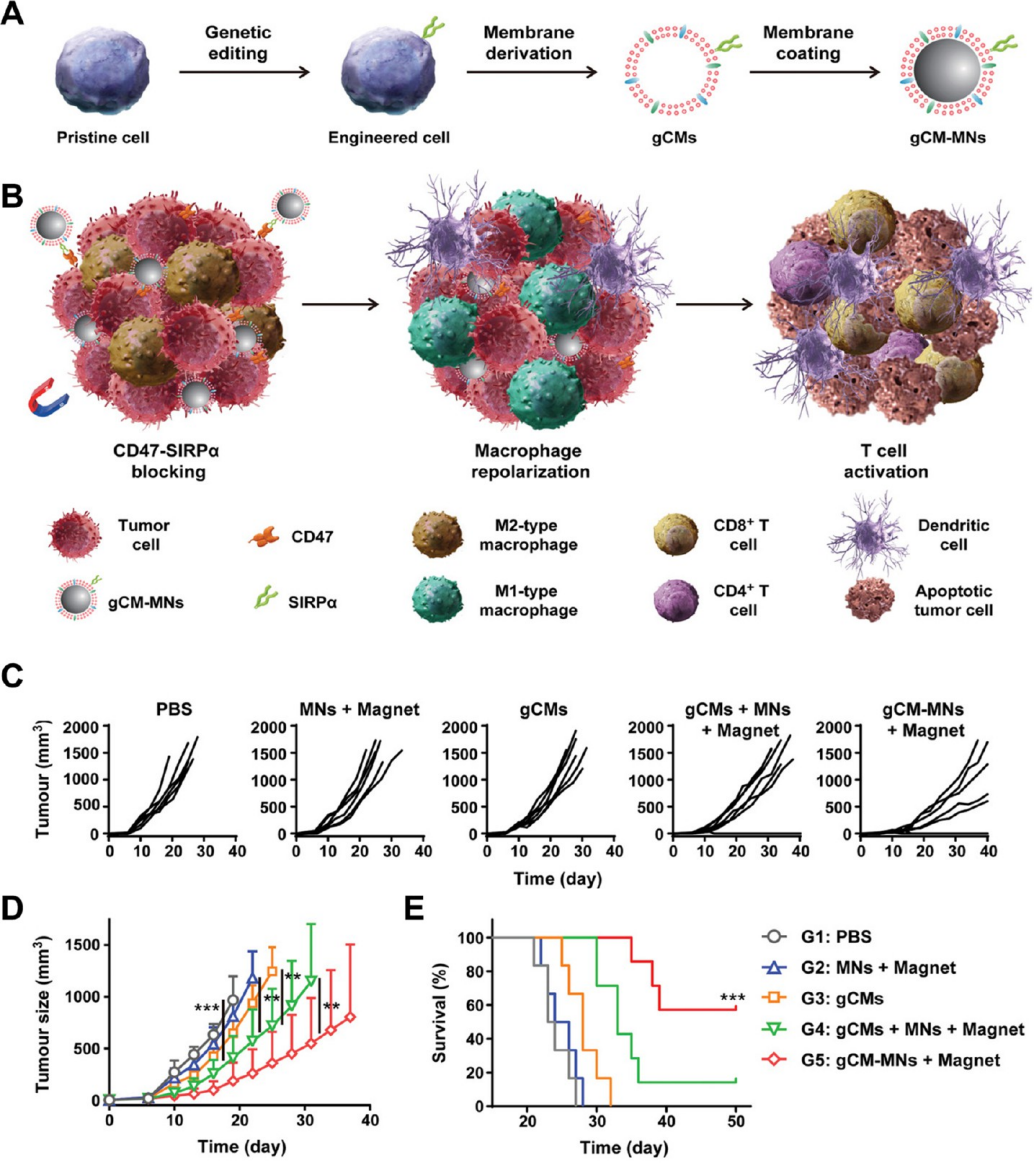
Schematics of magnetic nanoparticles coated with genetically modified cell membranes (gCM-MNs) for cancer immunotherapy. (A) Isolation and cell membrane coating. (B) Mechanisms demonstrating antitumor immunity. (C–E) The outcomes of in vivo anticancer activity. Reproduced from ref (64). Copyright 2020 Wiley.

The stimulation of macrophages for the purpose of cancer immunotherapy through the process of immunomodulation has surfaced as an extremely encouraging approach to treatment. However, activating macrophages effectively for anticancer immunotherapy faces two significant obstacles. First, the binding of signal regulatory protein (SIRP) to cluster of differentiation 47 (CD47), which is a “do not eat me” signal on cancer cells, inhibits the phagocytosis of malignant cells. Second, tumor cells secrete colony-promoting chemicals that polarize tumor-associated macrophages (TAMs) to a tumorigenic M2 state. It was discovered that genetically modified T cell membrane-coated Fe_3_O_4_ magnetic NPs (gCM-MNs) can overcome both these challenges. The gCM shell inhibits the CD47-SIRP pathway by genetically boosting the production of SIRP variants with extraordinary affinity, whereas the core of magnetic NPs triggers the repolarization of M2 TAM, resulting in combined macrophagic immune reactions. Moreover, the gCM coating shields the MNs from immune clearance, while the core of the magnetic NPs carries the gCMs to cancerous tissues through magnetic guidance, which ultimately amplifies their systemic circulation and accumulation in tumors (as illustrated in Figure 5A,B). In vivo anticancer effects were investigated using B16F10 mice carrying tumors. The therapy using gCMs-MNs considerably suppressed tumor progression in comparison with the treatments with magnetic NPs and gCMs. Furthermore, it exhibited greater efficacy than the combined delivery of gCMs and magnetic NPs (as depicted in Figure 5C–E). In melanoma and breast cancer models, gCM-MNs were shown to extend overall lifespan by inhibiting local tumor development and distant tumor dissemination. Hence, the use of genetic engineering and nanotechnology to activate the immune system for cancer immunotherapy is a safe and efficient approach.^64^

Type I interferons (IFNs) play a crucial role in regulating the interaction between tumors and the immune system. Patients whose IFN signaling is disrupted have a bad prognosis. Current IFN augmentation treatment can result in significant side effects, particularly IFN-induced multigenic resistance to immune checkpoint blockade (ICB). A solution to the problem is through the use of T cell membrane-coated IFN-epigenetic nanoinducer (OPEN) to mitigate the paradoxical consequences of IFN supplementation therapy. Scientists accomplished this method by modifying a cytotoxic T cell line to overexpress programmed death receptor 1 and employing their membrane to encapsulate protein NPs that carried ORY-1001, a lysine-specific histone demethylase 1 (LSD1) inhibitor. Following intravenous treatment, the OPEN facilitated the accumulation of ORY-1001 within tumors and production of IFNs, which subsequently stimulated the infiltration, activation, proliferation, and presentation of tumor-specific cytotoxic T cells and tumor cell antigen. Additionally, OPEN could easily block IFN-triggered programmed death ligand 1 and other immunological checkpoint molecules. This step-by-step technique reinstated intratumoral IFNs and decreased IFN-triggered immune dodging, which, in turn, diminished tumor growth in animal models. By utilizing an epigenetic nanoinductor, this study proposes a practical method to address the conflicting effects of IFN supplementation. This breakthrough in nanomedicine provides a safe and effective cancer immunotherapy.^65^ Overall, T cell membrane-camouflaged NPs hold great promise for enhancing cancer immunotherapy.

### 4.3 Photothermal Therapy

Thermal therapy using light, also known as photothermal therapy (PTT), offers several benefits as a promising cancer treatment.^66,67^ These advantages include improved effectiveness, noninvasiveness, and reduced harm to healthy tissues.^68^ The technique utilizes the ability of nanomaterials to convert optical light into heat energy and eliminate cancerous cells. However, the insufficient deposition of nanomaterials at the tumor site has impeded the use of nanotechnology in PTT.^69^ To address this challenge, the utilization of NPs coated with T cell membranes has surfaced as a hopeful nano-PTT for the purpose of targeting and eliminating tumors. This is because of their capability to augment the accumulation of nanomaterials and, hence, the efficiency of PTT.^70^

**Figure 6:**
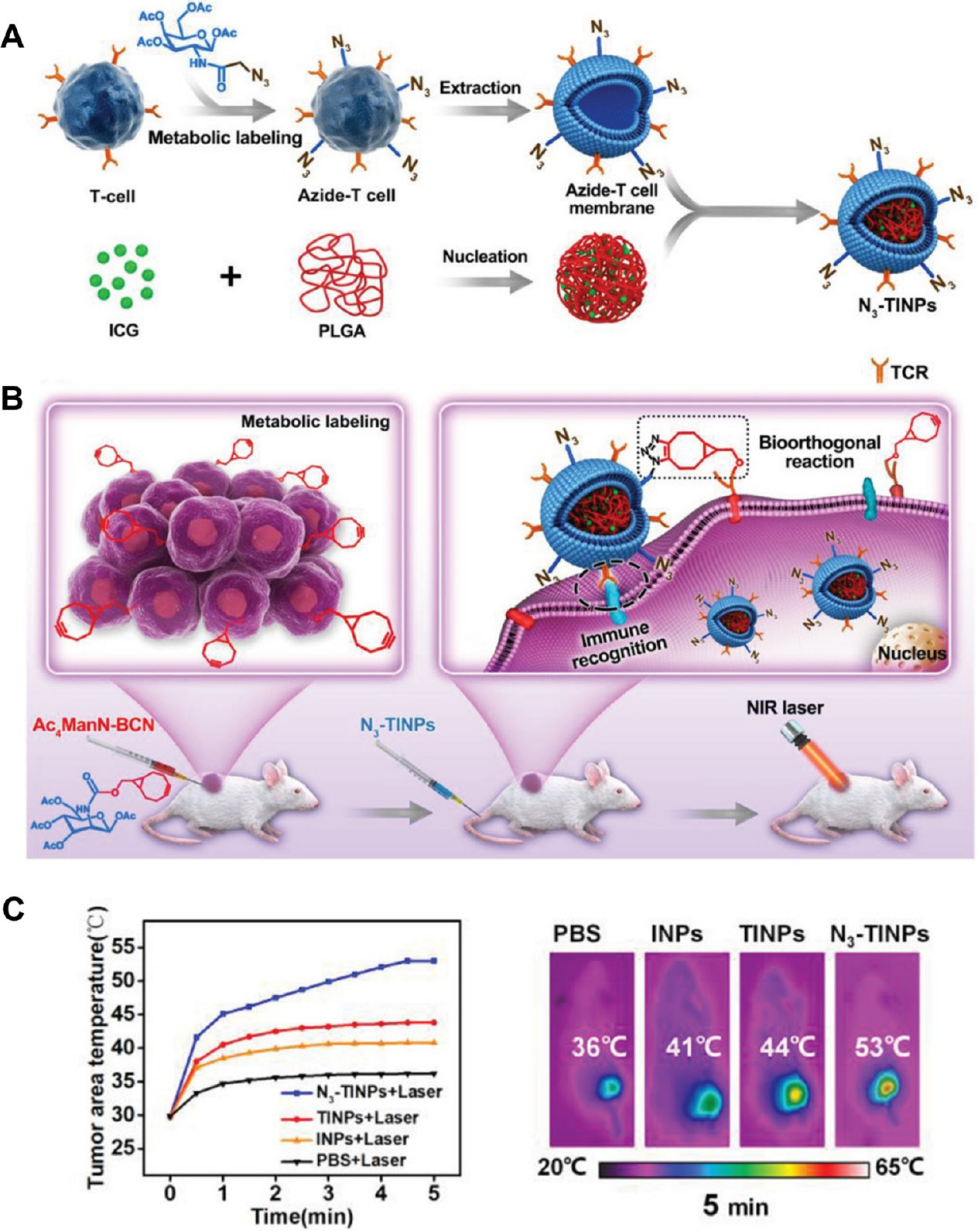
Nanoparticles that mimic T cell membranes and have dual targeting abilities are being used for photothermal treatment with great efficacy. (A) Production of N_3_-TINPs. To create N_3_-TINPs, T cell membranes labeled with N_3_ were extracted and coated onto ICG-PLGA polymeric cores. (B) Cancer cells that have the BCN group are targeted by N_3_-TINPs through immunological recognition of T cell membranes and bioorthogonal interaction between BCN and N_3_ groups, which leads to successful eradication of tumors in mice via ICG-mediated photothermal effects. (C) The effectiveness of photothermal therapy is demonstrated in vivo. Reproduced with permission under CC-BY license from ref (71). Copyright 2019 Wiley.

Recently, there has been extensive documentation on a personalized targeting strategy that is based on metabolic glycoengineering using bioorthogonal methods. The manipulation of artificial monosaccharides is an effective approach in adding diverse chemical groups to cell glycan through metabolic glycoengineering, enabling the specific production of bioorthogonal groups on tumors, which function as artificial “receptor-like” targets and can be utilized for targeted binding even in complex situations. An unnatural sugar, Ac_4_ManN-BCN, was chemically modified with bicyclononyne (BCN) and integrated into the surface glycans of tumor cells without causing damage. The BCN component on the membrane served as an excellent targeting label and significantly enhanced the recognition of tumors. Through this synthetic targeting technique, indocyanine green (ICG) NPs with T cell membrane coating (N_3_-TINPs) were created, which target tumor receptors. The findings indicated high tumor fluorescence intensity. Moreover, the PTT efficacy of N_3_-TINPs was evaluated in vivo, and the temperature of the tumor area in the TINPs group increased to 44 °C. In contrast, it rose significantly to 53 °C in the N_3_-TINPs group (Figure 6).^71^

**Figure 7:**
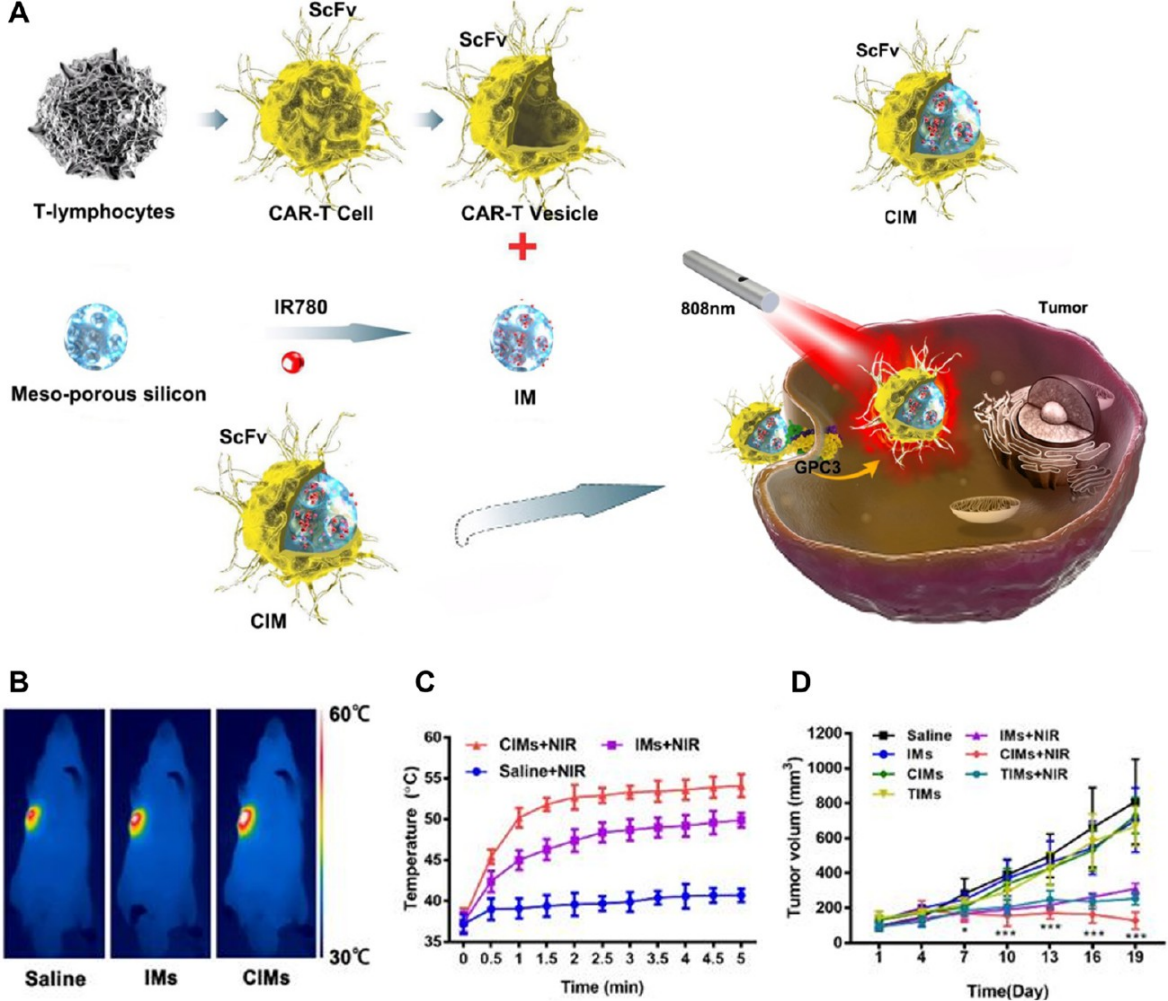
Nanoparticles coated with CAR-T membrane have been utilized for antitumor therapy through photothermal applications. (A) The use of CAR-T membrane to coat nanoparticles has been illustrated to exhibit photothermal action against tumors. (B–D) The efficacy of this approach has been demonstrated in vivo. (B) NIR resulted in a temperature increase in tumor-bearing Huh-7 nude mice, as shown in infrared thermographic images. (C) Various NIR irradiation treatments were administered through intravenous injection, which resulted in different temperature rises. (D) Tumor development profiles were also monitored. Reproduced with permission under CC-BY license from ref (70). Copyright 2020 The Authors.

Recently, a new type of nanomaterial that combines the benefits of NP drug delivery technology with the targeting ability of chimeric antigen receptor (CAR)-T cells was proposed. This innovative approach involves using T cell membrane-coated mesoporous silica NPs loaded with IR780 dye to achieve highly efficient PTT anticancer properties and improved targeting capabilities. The physical properties of the NPs were analyzed, and their targeting capabilities were confirmed. TEM verified that NPs were successfully deposited on the cell membrane. The NPs coated with CAR-T cell membrane displayed better targeting efficacy compared with the unaltered NPs (Figure 7). This strategy demonstrated enhanced targeting of hepatocellular carcinoma (HCC) via the utilization of a T cell membrane coating approach, which opens up new directions in the treatment of HCC.^70^

### 4.4 Combinatorial Therapy

Because of the diversity of cancerous diseases, a solitary treatment method may not be adequate. Hence, a fusion therapy approach can prove to be more efficacious in eradicating cancerous growths. The combinatorial therapy encompasses the amalgamation of two separate treatments, namely radioimmunotherapy, chemoradiotherapy, or a pharmaceutical blend that can aim at various cancerous growth pathways.^72^

The combined use of chemotherapy and radiation, known as chemoradiation, has developed into a crucial treatment method for effectively managing various types of solid tumors in the last 30 years. Despite its success, this strategy has several drawbacks. The initial tumor cannot always be eradicated by chemoradiotherapy. In addition, the combined application of chemotherapy and radiation therapy has failed to decrease the radiation dosage required for a successful treatment outcome. Moreover, it has substantially augmented the adverse effects associated with cancer treatment.^73,74^

The innovative advancement of NPs camouflaged with T cell membranes enhances the administration of chemotherapy. As a result, the potency of chemoradiotherapy is enhanced with reduced toxicity. To achieve this outcome, a nanotechnology system that mimics biological processes, composed of membranes from cytotoxic T lymphocytes, was presented. The exterior of PLGA NPs was concealed with T lymphocyte membranes. To target the NPs, a local low-dose irradiation (LDI) treatment was utilized as a chemical lure. Using this innovative method, NP phagocytosis by macrophages was reduced by 23.99% (*p* = 0.002). Balb/c nude mice treated systemically with paclitaxel-loaded T lymphocyte membrane-cloaked NPs impeded the cancer growth by 56.68%. When LDI was given to the tumor site, this value increased to 88.50%, and two animals experiencing full recovery. In addition, LDI can enhance the stimulation of adhesion molecules in tumor vasculature, which is required for leukocyte attachment and may help in the tumor localization of T lymphocyte membrane-coated NPs. This method of administering drugs maintained the effectiveness of human cytotoxic T cells, extended their time in circulation, and enhanced their accumulation at the tumor site.^75^

**Figure 8:**
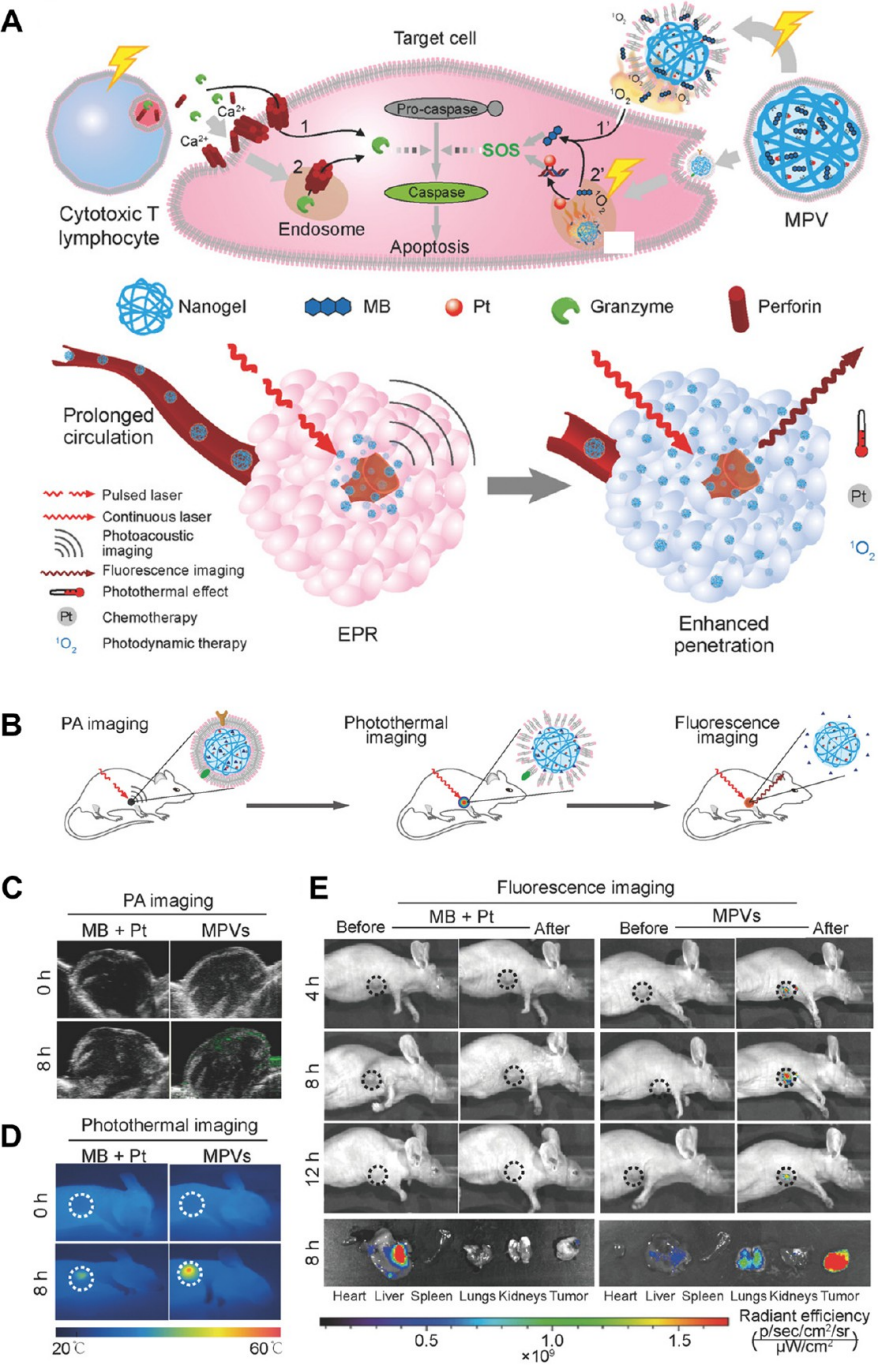
Combinatorial treatment of nanoparticles coated with T cells. (A) Depiction and mechanism of MPVs. (B) Diagrammatic representation of the tracing of inexperienced, stimulated, and active MPVs in mice with 4T1 tumors using fluorescence imaging, photoacoustic, and photothermal techniques. (C) Photoacoustic imaging of the gathering of MPVs within tumors at 0 and 8 h following injection. (D) The infrared thermographic images of rodents that underwent treatment with MPVs and localized irradiation. (E) Typical fluorescence images of tumor-affected mice after PTT. Reproduced from ref (77). Copyright 2018 Wiley.

The utilization of chemotherapy and PTT has emerged as a hopeful approach for dealing with cancer. Nevertheless, obstacles, such as targeted administration and drug release at specific tumor locations, need to be overcome.^76^ Consequently, a platform that can convey both chemotherapy and PTT agents to the affected area is necessary for the combined treatment of different types of tumors. To achieve this, Zhai et al. designed nanovesicles (MPVs) with a core loaded with cisplatin (Pt) and methylene blue (MB) and coated with a cell membrane. The MPVs produced contrast for tumor photoacoustic imaging and induced hyperthermia, thereby enabling PTT activity and infiltration of tumor. The merge of PTT, PDT, and chemotherapy resulted in tumor reduction and a 97% reduction in lung metastasis (Figure 8). The distribution and activation of MPVs were assessed in tumor-bearing 4T1 mice after intravenous delivery using photoacoustic, PTT, and fluorescence imaging. Additionally, the MPVs achieved great tumor penetration and efficient lysosomal escape, which resulted in regression of the main tumors and an ∼98% suppression of pulmonary metastasis. The MPVs demonstrated tumor selectivity and aggregation, stronger tumor infiltration, and concurrent triple therapy. Consequently, they were identified as a hopeful nanotherapy for the management of metastatic breast cancer.^77^

Chemotherapy and immunotherapy are combined in chemoimmunotherapy to prevent the formation, spread, and recurrence of tumors. However, challenges remain in administering therapeutic substances to target locations, which can result in unexpected levels of drug in tumor. Additionally, the majority of immunotherapeutic medications are susceptible to decay caused by enzymes and chemicals, which leads to a decrease in their biological efficacy.^78^ To create a successful combination treatment, a biomimetic nanocarrier is necessary to load and transport both drugs simultaneously. Dual-responsive NPs were produced by combining hyaluronic acid–disulfide bond–vitamin E succinate with curcumin and coating them with a modified T cell membrane to create RCM@T. T cell membrane protected medication delivery and served as a programmed cell death-1 antibody to specifically bind tumor cells. After intravenous treatment, RCM@T amassed at acidic tumor sites and demonstrated a “membrane escape effect,” thereby revealing the hyaluronic acid component for delivering drugs that target tumors specifically. The cellular particles amassed in the cytoplasm via CD44-facilitated internalization and liberated the loaded curcumin inside the cell in an oxidative microenvironment. Debris from T cell membranes targeted the PD-L1 of cancerous cells for the purpose of tumor immunotherapy, thereby directly eliminating tumor cells, intensifying the CD8^+^ T cell count, and inciting the release of cytokines. RCM@T offered an innovative approach for designing rational anticancer nanodelivery systems by combining responsive drug release, delivery of chemotherapeutic agents, and immune checkpoint blockade immunotherapy on the basis of cell membranes.^79^

The latest progress in creating NPs coated with T cell membranes is poised to bring about a significant breakthrough in the application of combination therapy to tackle the difficulties of creating effective novel medications. This will ultimately enhance the outlook for the use of combination therapy cancer treatment.

## 5 Viral Infectious Diseases

Cell membrane-coated NPs have shown strong efficacy in antiviral therapy recently. They can serve as decoys to counteract pathogenic viruses in plasma, thereby redirecting pathogens away from their intended target cells. However, NPs coated with cell membranes have the ability to regulate the persistence of viruses in host cells that have a lengthy lifespan. Therefore, the utilization of cell membrane-coated NPs might serve as an effective means to eliminate viruses in both plasma and persistent cell reservoirs. RNA viral infections, including HIV, Zika virus (ZIKV), and SARS-CoV-2, present substantial risks to human health. The utilization of combination antiretroviral therapy is wide-ranging, but the emergence of resistance and the difficulties associated with long-term medication pose significant challenges to traditional therapies. Cell membrane-coated NPs possess prospects in antiviral therapy.^80^

Despite recent advancements in treatment, HIV type-1 infection is still fatal. Eliminating the virus is challenging because of the presence of residual viruses that evade therapy. Although the current antiviral regimen can control plasma virus levels at an extremely low level, it must be taken continuously because viral reactivation can occur quickly after therapy withdrawal.^81^ Even with combination antiretroviral therapy, residual cells continue to harbor the virus, thereby resulting in active viral replication in dormant cells. Moreover, traditional pharmacological therapy’s effectiveness is restricted by its adverse reactions and the emergence of antimicrobial resistance. Antibodies that aim to neutralize the glycoproteins on the surface of circulating HIV particles are widely researched as a potential remedy for these issues.^82^ However, because of the limited immunity these antibodies generate, their ability to neutralize free viruses is insufficient, and the therapy’s effectiveness remains unsatisfactory.^83^ To prevent HIV infection, there has been a significant effort to develop secure and effective vaccines.^84^ However, as of yet, no immunogen for the HIV envelope (Env) has been discovered that can generate antibodies with broad neutralizing activity. Clearly, there is an urgent requirement for inventive treatments to combat HIV infection. Recently, therapeutic nanomaterials have been developed to improve the prevention and management of HIV.^85^ NPs have been utilized as delivery systems to increase the efficacy of antiviral drugs. Moreover, certain NPs have been found to obstruct viral assembly and impede reproduction by utilizing methods like the simultaneous manifestation of multiple molecules and direct inhibition.^86^ Vaccines utilizing NPs also hold considerable promise in altering host immune responses through better immunological targeting and the fusion of antigen and adjuvant expression. This collaboration enhances safety and anti-HIV immunity. NPs coated with cell membrane have surfaced as a new biomimetic approach to manage different human ailments.^87^ The biological characteristics of donor cells can be emulated by NPs using synthetic cores that are enclosed with natural cellular membranes. By using cell membrane-cloaked NPs, it is possible to trick vulnerable cells into eliminating pathogens. The effectiveness of RBC-NPs in neutralizing pathogenic autoantibodies, neurotoxins, and bacterial pore-forming toxins has been well-documented.^88^ Likewise, NPs enveloped with macrophage membranes can counteract endotoxins and inflammatory cytokines. After the preliminary stage, NPs enveloped with membranes derived from diverse cell categories, such as malignant cells, thrombocytes, white blood cells, stem cells, and microorganisms, have been triumphantly fabricated. These NPs provide extensive therapeutic potentials because of their cell-like traits and intricate biointerfacing.^89^ Consequently, the exceptional biomimetic ability of NPs enveloped in cellular membranes has resulted in the innovation of this technique for potential HIV therapy.

**Figure 9:**
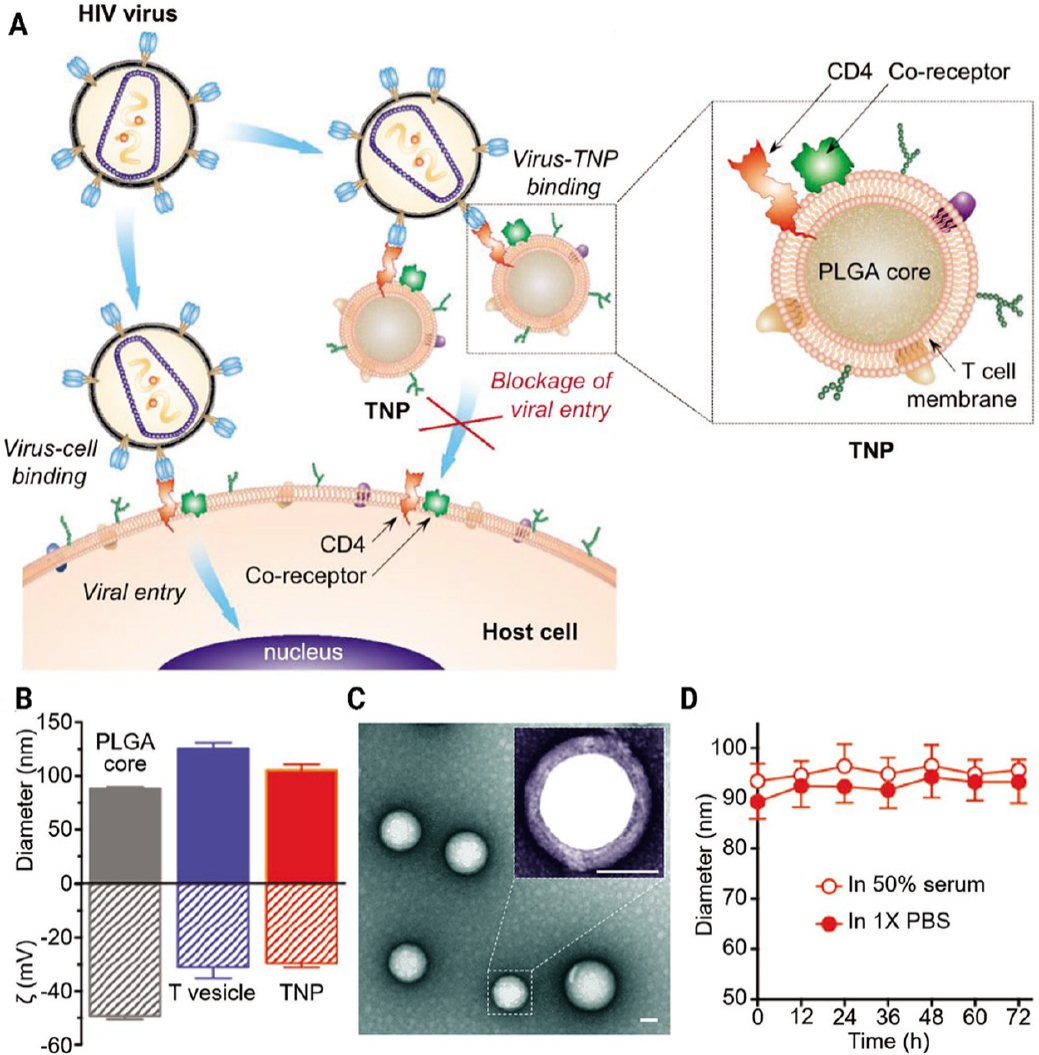
Schematic representation of T cell membrane-coated NPs and physicochemical characterizations. (A) Illustration of TNPs. (B) Determination of size and hydrodynamic size. The deviations from the mean values are shown by error bars (*n* = 3). (C) Images of TNPs observed through transmission electron microscopy and stained negatively with uranyl acetate. The scale bar represents 50 nm. Inset: a closer view of an individual TNP. Scale bar = 50 nm. (D) The stability of TNPs was assessed by measuring the size of particles for a duration of 72 h. Reproduced from ref (90). Copyright 2018 Wiley.

Wei et al. undertook a study that was inspired by the latest developments in cell membrane coating technology.^90^ They synthesized T cell membrane-coated NPs (TPNs) by wrapping CD4^+^ T cell plasma membranes around polymer cores (Figure 9). The TPNs inherited the accompanying CD4 receptor and CCR5 or CXCR4 coreceptors, in addition to other T cell surface antigens that are required for HIV attachment. The TPNs act as a camouflage for viral assault to redirect the viruses away from their original host targets, thereby ultimately eradicating HIV. The strategy employed by this decoy technique imitates the actions of host cells to inactivate viruses, as opposed to directly aiming at the viral replication mechanism. Furthermore, it possesses the capability to conquer the genetic variability of HIV. The research indicated that TPNs have a distinct affinity for gp120, a vital glycoprotein present on the surface of HIV, and effectively halt gp120-triggered CD4^+^ T cell apoptosis. TPNs effectively combat the viral infiltration of human macrophages derived from monocytes and mononuclear blood cells after being exposed to HIV. TPNs, utilizing inherent T cell properties, possess substantial promise as a novel treatment for HIV infection.

A related study investigated the possibility of using CD4^+^ T cell NPs that were covered with a membrane (TNPs) to combat different strains of HIV-1.^91^ TNPs demonstrated extraordinary ability to neutralize and cover a wide range of viruses, with an average IC_80_ of 819 g/m. They efficiently eliminated all 125 HIV-1 pseudotyped viruses that were examined, which encompassed recombinant types and transmitted/founder viruses. TNPs did not have any impact on uninfected cells, but selectively adhered to and stimulated autophagy in HIV-1-infected macrophages and CD4^+^ T cells. This autophagy, which was induced by TNPs, suppressed HIV-1 that was associated with cells and impeded viral release in a dose-dependent manner that was dependent on phospholipase D1. This impact was mitigated by inhibiting autophagy pharmacologically or genetically. TNPs can serve as a therapeutic drug to decrease the HIV-1 reservoir by neutralizing cell-free HIV-1 and targeting HIV-1 gp120-expressing cells.

Zika virus, discovered in 1947, is a mosquito-borne flavivirus that causes mild symptoms in humans. Since 2015, it has spread to over 20 countries and become a global public health issue. The virus is linked to neurological complications, including microcephaly in fetuses, Guillain–Barré syndrome, meningoencephalitis, and testis damage in mice. Despite progress, no licensed vaccine or treatment is available. A study by Rao et al. presents an anti-ZIKV host-mimicking nanodecoy (ND) that uses biomimetic cell membranes to conceal nanomaterials.^92^ ND wrapped by a polymeric core can improve the survival and retention of ZIKV in the bloodstream by stabilizing the host cell membrane shell. This structural feature effectively diverts ZIKV from its intended targets. NDs can inhibit ZIKV replication in vitro, abolish inflammatory and degenerative changes in mice, and suppress fetal microcephaly. Biomimetic NDs exhibit superior biocompatibility, effective immune evasion, and long circulation time, which makes them a promising antiviral application in the treatment of various diseases caused by ZIKV.

The COVID-19 pandemic, caused by the highly transmissible SARS-CoV-2, poses a global threat to public safety. Despite effective therapies, such as inhibitory drugs and vaccines, tackling the broad spectrum of coronavirus is a long-term task, which necessitates understanding of the pathogenic mechanism for potential therapeutic tools.^93^ The SARS-CoV-2 virus’s entry into host cells is mediated by the viral spike glycoprotein (S protein), which has two subunits for natural cell recognition and fusion. The S protein’s attachment to epithelial cells triggers downstream immunological effects, which leads to respiratory failure, the main cause of death in COVID-19 patients. Zhang et al. presented cellular nanosponges as a potent medicinal intervention against the SARS-CoV-2 virus in a study.^94^ There are two categories of cellular nanosponges, which are composed of plasma membranes obtained from either human lung epithelial type II cells or human macrophages. These nanosponges possess the identical protein receptors, including both known and unknown ones, that are necessary for the cellular penetration of SARS-CoV-2. Evidence demonstrated that after being exposed to the nanosponges, SARS-CoV-2 was effectively neutralized and lost its ability to infect cells. Importantly, the nanosponge platform was not affected by viral mutations and had the ability to target various viral species. The nanosponges possessed the ability to kill the virus as long as they continued to target the identified host cell.

In previous sections, cellular membrane-coated NPs developed for virus-based diseases were mentioned. However, there are very few studies in this field where no therapeutic study exists that uses T cell membrane-coated NPs that were created to treat virus-related diseases like ZIKV and SARS-CoV-2. Future studies in this field, in order to close this gap in the literature, will have promising potential impact.

## 6 Natural Killer Cell Membrane-Camouflaged Nanoparticles

**Figure 10:**
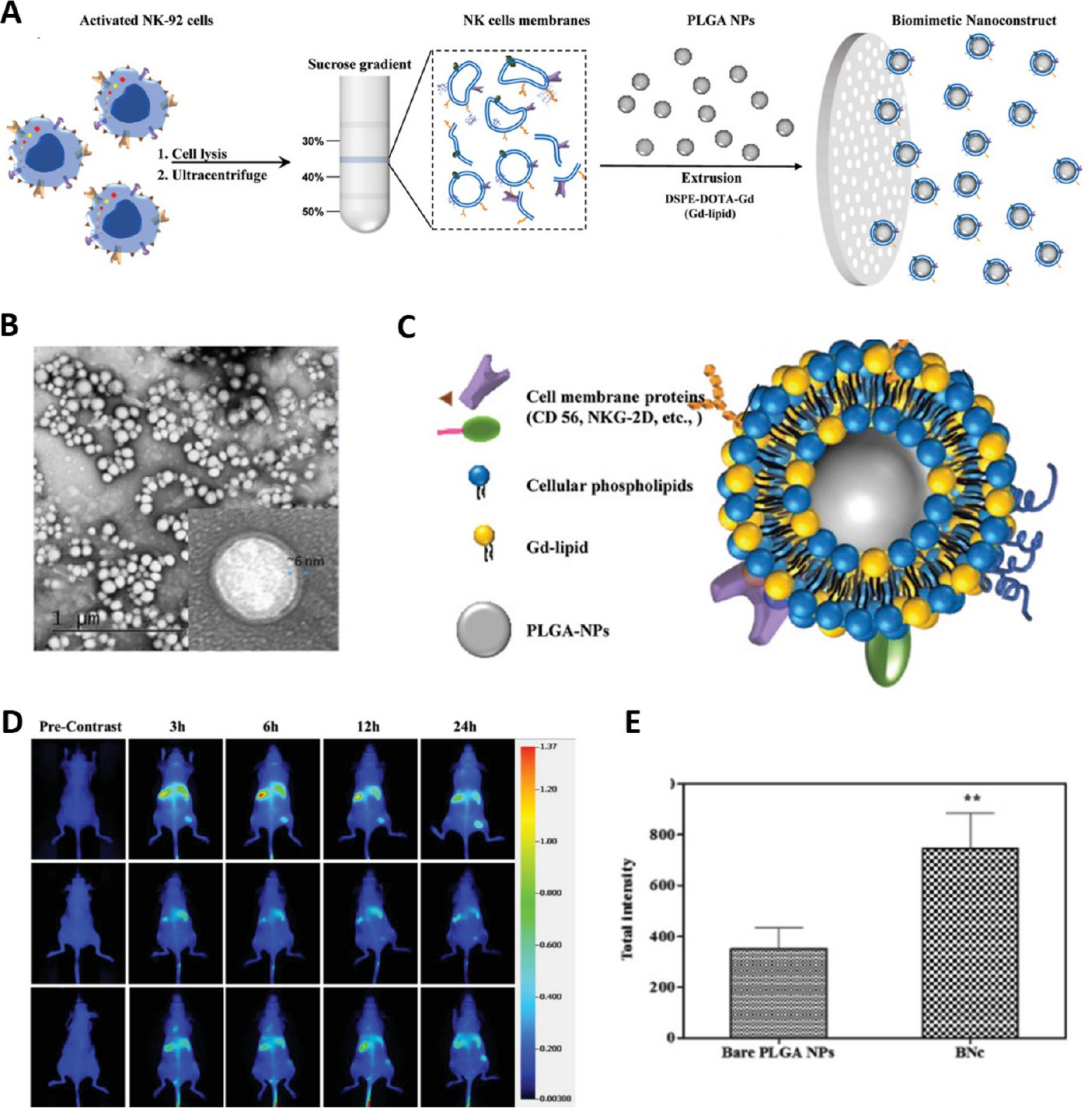
Biomimetic liposomes loaded with natural killer cells for targeted tumor therapy. (A) The process of producing biomimetic nanoconstructs involves a schematic depiction of natural killer cell membrane separation. Subsequently, biomimetic nanoconstructs were produced through a straightforward extrusion method. (B) Transmission electron micrograph of the nanoconstructs; inset depicts an enlarged picture of the characteristic biomolecular corona (NKM and Gd-lipid). (C) The structural content of nanoconstructs. (D) Dynamic real-time observation of NU/NU mice with MCF-7 tumors after receiving nanoconstructs labeled with DiR at a dose of 10 mg/kg through intravenous administration. Images were captured at 3, 6, 12, and 24 h before injection. (E) Assessment of the buildup of nanostructures and PLGA nanoparticles in NU/NU mice with MCF-7 tumors 24 h following administration. Reproduced from ref (96). Copyright 2018 Wiley.

Natural killer cells (NK cells) are a crucial type of immune cells in the human body that are essential for defending against cancer and infections. They can trigger cell death by releasing cytotoxic chemicals or by binding to certain receptors. NK cells can eliminate tumor cells by utilizing RANKL and DNAM1 proteins on their membrane, independent of tumor antigens. Membrane proteins like IRGM1, CB1, and Galectin12 have the ability to alter macrophages to combat malignancies.^95^ Lately, there has been an increase in interest toward a revolutionary technology referred to as cell membrane-cloaked NPs. Among numerous mammalian cell membranes, the natural killer cell membrane (NKM) has proven to be a promising coating for NPs that aims to address challenges associated with targeting ability, residence duration, biocompatibility, biodistribution, and resistance of anticancer drugs.^95^ The use of natural killer cells from mammals to cloak synthetic drug delivery NPs in a biomimetic manner has garnered significant interest. This approach has shown promise in delivering anticancer drugs to specific targets by incorporating the biological intricacy of the cells. As a result, researchers have developed NKM-camouflaged NPs. In a recent study, a biomimetic nanoplatform was created by coating carboxylate-terminated polylactic-*co*-glycolic acid biomimetic nanoconstructs with NKM camouflage. Gadolinium contrast agents and near-infrared (NIR) dyes were then integrated into the nanoconstructs, and their imaging abilities were evaluated using MRI and NIR fluorescence. By modifying the density of Gd-lipid conjugate on the exterior of the nanostructure, the magnetic characteristics could be finely adjusted. The NKM-camouflaged system displayed distinct interaction with MCF-7 cells compared with the plain polymeric NPs, as revealed by confocal imaging and cell sorting investigations. These findings underscored the tumor-targeting potential of the biomimetic nanoconstructs. Validation of this cellular interaction was further confirmed through in vitro tests, in vivo tumor imaging using NIR fluorescence, and ex vivo MR imaging (Figure 10).^96^

**Figure 11:**
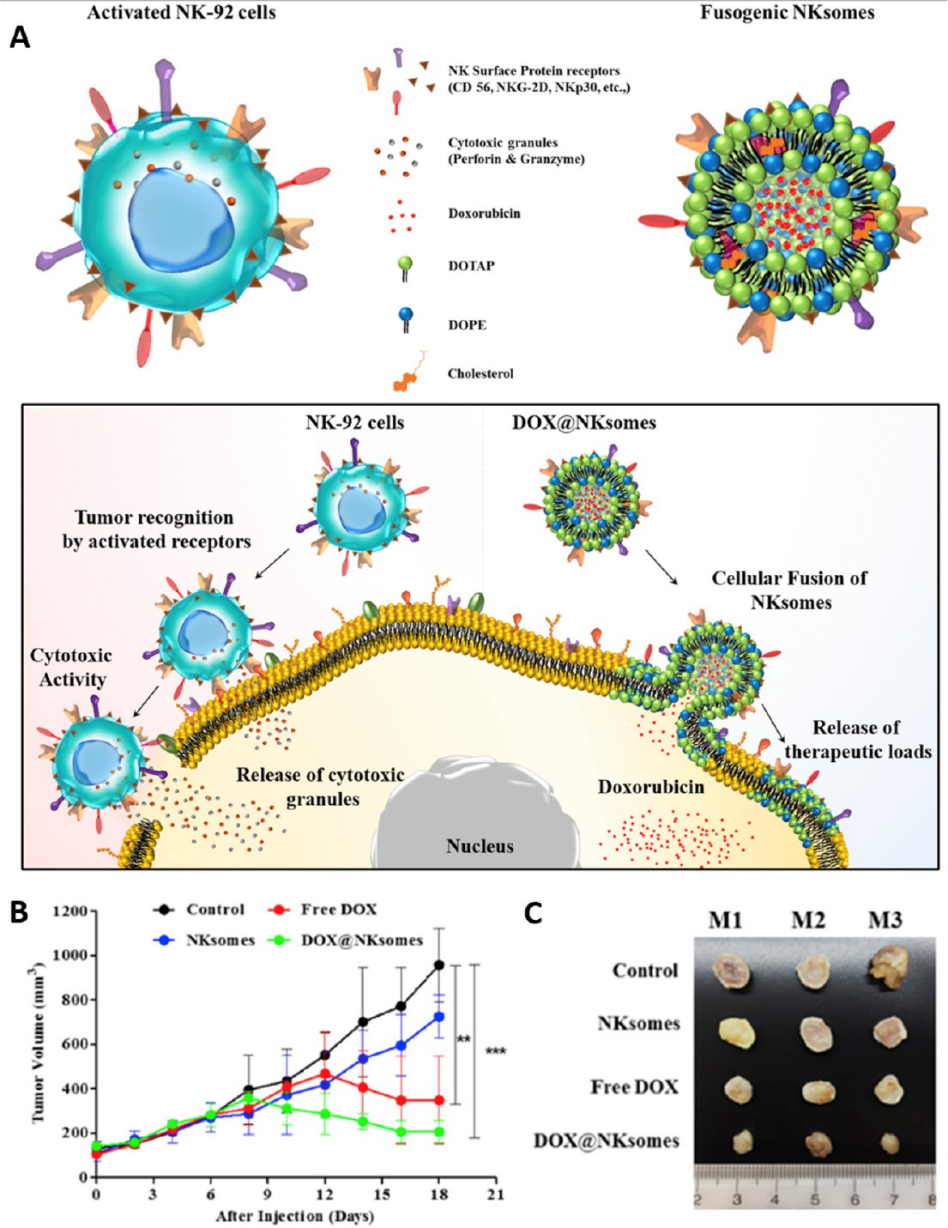
Biomimetic liposomes infused with natural killer cells are utilized for targeted tumor therapy. (A) The diagram illustrates the utilization of activated NK cells and their membrane-derived fusogenic liposomes (known as NKsomes) for targeted tumor therapy. By detecting elevated surface stress signals, NK cells can promptly differentiate cancerous cells and release cytotoxic granules to activate its antitumor potential. Similarly, DOX-carrying NKsomes can detect cancerous cells by utilizing NK cell markers and merge more efficiently with these cells than with healthy ones, therbey indicating their potential as an antitumor agent through the release of DOX. (B) The tumor volume change in mice who received free DOX (5 mg/kg), DOX-loaded NKsomes (with an equal DOX concentration of 5 mg/kg), bare NKsomes (10 mg/kg), and untreated control groups. DOX@NKsomes successfully hindered tumor growth in NU/NU nude mice with MCF-7 tumors and weakened immune systems. (C) Tumors at the end of the therapies. Reproduced from ref (31). Copyright 2018 Elsevier.

In the subsequent examination, a biomimetic nanoarchitecture (NKsome) made up of fusogenic liposomes infused with NKM was created. The receptor proteins containing activated NKM were separated from NK-92 cells and combined with the fusogenic liposome to form NKsomes. These were found to be physically stable, capable of loading doxorubicin (DOX), and nonimmunogenic, thereby making them ideal for targeted cancer therapy. Additionally, pharmacokinetic and biodistribution studies indicated that NKsomes have an extended half-life in circulation and an increased potential for homing in on tumors. The effectiveness of NKsomes in targeting cancer was assessed through in vitro experiments using normal osteoblasts (NHost) and human breast cancer cells (MCF-7). Following treatment, mice that received free DOX and DOX@NKsomes experienced a noteworthy decrease in the volume of the tumor, while bare NKsomes facilitated tumor growth (as shown in Figure 11). This investigation demonstrated the tumor-targeting ability of NKsomes for precise cancer therapy by leveraging the unique characteristics of natural killer cell membranes. This could pave the way for novel biomimetic nanomedicine design considerations.^31^ The revealed immunosurveillance capabilities of these biomimetic nanoplatforms indicate that NKM-coated NPs offer promise for medication administration by boosting the diagnostic efficacy of targeted cancer bioimaging.

One of the primary hurdles in treating cancer is the emergence of resistance to multiple drugs, known as multidrug resistance (MDR). Fortunately, research has shown that natural killer (NK) cells can enhance the permeability of the tumor plasma membrane, thereby allowing granzymes to penetrate and eliminate target cells. Building on this discovery, Li et al. developed a biomimetic nanosystem that can increase the amount of therapeutic drugs within tumor cells. The NK cell-biomimetic NPs (DMLN) were designed to surmount MDR by catalyzing the entrance of DOX into tumor cells. The DMLN nanosystem was composed of NKM and hollow MnO_2_ NPs loaded with lactate oxidase/DOX. When the NPs reached the tumor site, they released OH radicals through a Fenton-like reaction, which improved the permeability of the tumor plasma membrane and facilitated the entrance of DOX into the cells. This, in turn, increased the intracellular drug concentration and effectively reversed the MDR. Excellent antitumor efficacy of DMLN in drug-resistant tumors was proven. Additionally, the release of Mn^2+^ from the NPs makes it possible to use them for superior MRI imaging in vivo. Taken together, this biomimetic nanoplatform that combines NKM with synthetic materials has enormous possibilities for surmounting tumor drug resistance.^97^

**Figure 12:**
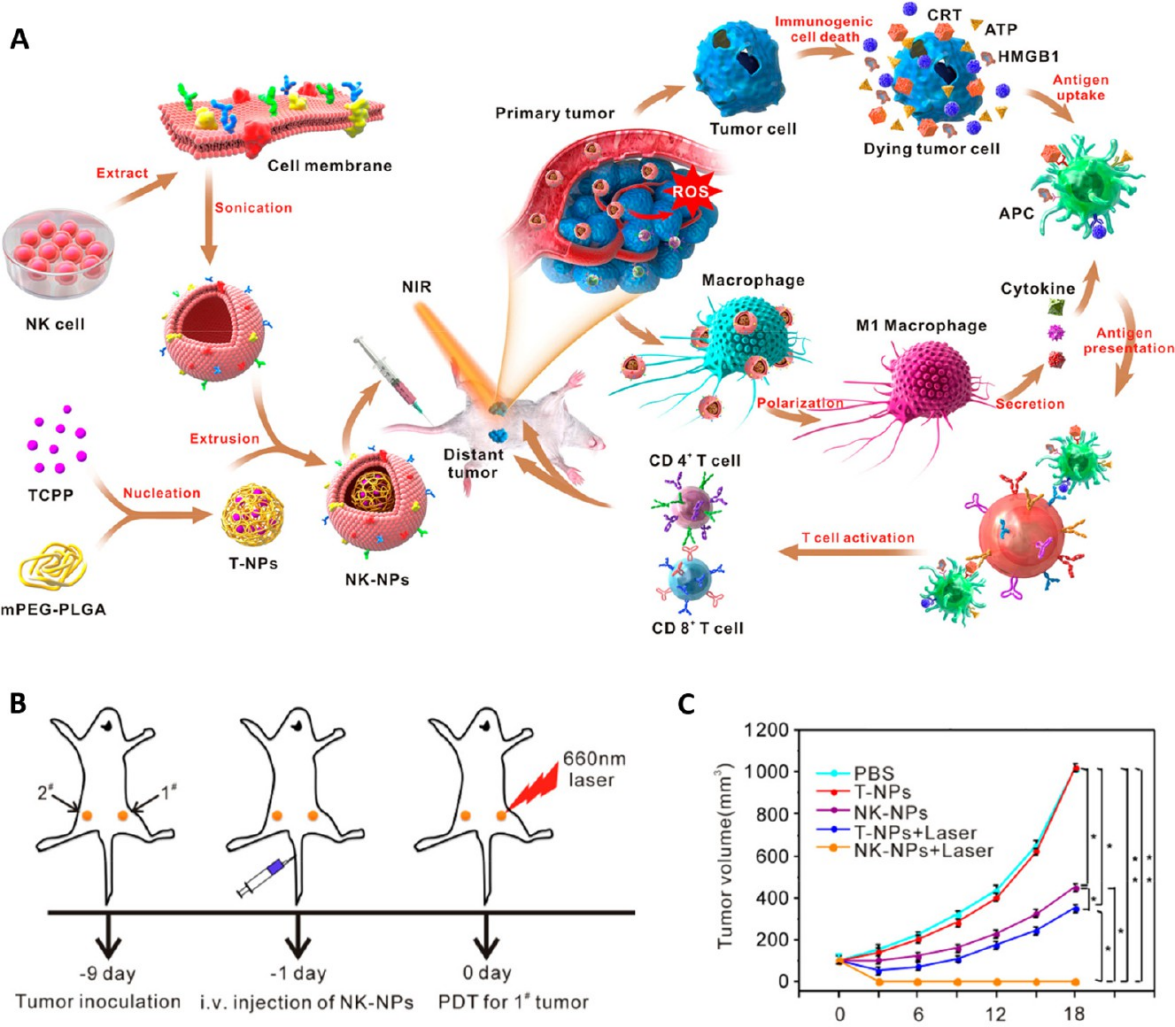
Nanoparticles coated with NK cell membrane (NKM) for enhanced immunotherapy through photodynamic therapy (PDT). (A) NKM was extracted and coated onto polymeric nanoparticles loaded with the photosensitizer tetra(4-carboxyphenyl)porphyrin (TCPP) through extrusion. NKM facilitated the ability of NK-NPs to polarize tumor cells to proinflammatory M1 macrophages, thereby leading to the production of cell membrane immunotherapy. NK-NPs induced immunogenic cell death (ICD) through PDT, causing dying tumor cells to produce damage-associated molecular patterns to enhance the NKM immunotherapy effect. Immunogenic PDT significantly improved NKM immunotherapy, which resulted in a substantial increase in the influx of effector T cells. (B) Schematic representation of the experimental setup. The tumors on the right side were treated with PDT and labeled as “primary tumors,” while the tumors on the left side were designated as “distal tumors” and were not treated with PDT. (C) Tumor growth curves. Reproduced from ref (100). Copyright 2018 American Chemical Society.

Cancer immunotherapy and photodynamic therapy (PDT) were discovered to be the largest scientific breakthrough in oncology.^98^ Nevertheless, the clinical implementation of these therapeutic approaches encounters obstacles because of the intricacy of tumors, diversity among patients, and the risk of systemic toxicity. Thus, the development of tumor-specific and long-lasting immune responses without causing harm to the body remains a challenge.^99^ To address this issue, Deng et al. proposed that NKMs could trigger M1 macrophage polarization and generate tumor-specific immune responses by targeting cancer cells. To achieve this goal, they designed NKM-coated NPs that could enhance the efficacy of immunotherapy and achieve the desired therapeutic effect in animals (Figure 12). Shotgun proteomics was used to profile the NKM, which enabled the coated NPs to target tumors and promote M1 macrophage polarization, thereby inducing an anticancer immune response. Besides, the NPs directly eliminated primary tumor cells by employing PDT. Additionally, these NPs induced the production of damage-associated molecular signals in dying tumor cells. As a result, the antigen-presenting cells were activated, and the efficiency of antitumor immunity was enhanced in natural killer cells. The findings of this study show that NKM-coated NPs preferentially accumulate in the tumor, therefore providing an alternative strategy to advance immuno-PTT in the near future.^100^

For cancer treatment to be effective, drugs must be delivered precisely to the site of action. Exosomes are endogenous nanovesicles capable of transporting biological information between cells. Every cell type, including immunological cells like NK cells, releases them. Nevertheless, mammalian cells secrete only a limited number of exosomes, and their isolation is a difficult task.^101^ Zhu et al. analyzed the exosome mimics (NK-EM) generated by NK cells and demonstrated their effectiveness in fighting tumors. The exosomes were generated by filtering NK cells through progressively smaller pores. The anticancer properties of NK-EM were evaluated against various types of cancer cells, including breast carcinoma, glioblastoma, anaplastic thyroid cancer, and hepatocellular carcinoma, using bioluminescence imaging (BLI) and the CCK-8 assay. In vivo, the antitumor effect of NK-EM was validated by substantial reductions in BLI, tumor size, and tumor mass compared with the control group. These findings indicate that NK-EM is more potent against cancer cells than traditional NK-exosome, and its capacity to target tumors has been confirmed in vivo. Therefore, NK-EM has the potential to be a promising immunotherapeutic drug for cancer treatment.^102^

NK cells can kill cancer cells directly by releasing cytokines and exerting their cytotoxic action without requiring prior exposure to the antigen, unlike T cells. NK cells are part of the innate immune system and can identify the target antigen without relying on major histocompatibility complex I (MHC-I).^103^ NK cells can identify and target cancer cells due to the modulation of NK cell receptors. Furthermore, T cells and NK cells have been modified to produce chimeric antigen receptors (CARs) that target tumors specifically, thereby enhancing their effectiveness and capabilities. T cells and NK cells have significant potential to facilitate medication delivery for cancer treatment.^104^

Cell membrane coating nanotechnology is the process of enveloping artificial nanoparticles with cell membranes from various cell types to give the NPs the characteristics of a particular cell type, which enhances the accuracy and efficacy of disease treatment. The origin of cell membranes can influence the functionalities of nanoparticles. Cell membrane vesicles and exosomes, which mimic biological structures, are frequently utilized as transporters.^19^ Cell membrane vesicles and exosomes share similar functions in biocompatibility and targeting when compared to cell membrane-coated NPs. Nevertheless, these biomimetic carriers have several drawbacks. Delivering hydrophobic medications, codelivering pharmaceuticals with various characteristics, and achieving controlled release can be challenging. Furthermore, the cell membrane yield is significantly greater than that of exosomes. Cell membrane-coated NPs possess the inherent properties of the original cells, along with the effective drug transport and targeted drug release capabilities of nanoparticle cores. This enables easier implementation of multifunctional design and fulfillment of diverse functional needs. The complex design of cell membrane-coated NPs may hinder their clinical translation.^105^ In this regard, membrane selection for coating is also very important. NK cells, which have advantages, such as tumor targeting and long-term circulation, can be selected as potential cell-covering membranes. However, disadvantages, such as the difficulty of cell membrane isolation and coating methods and the fact that these technologies are not yet fully developed, should be taken into consideration.^95,106^

## 7 Current Challenges and Future Directions for Clinical Translation

Although cell membrane-coated nanomaterials hold promise for various disease conditions, there remain certain obstacles linked to this technology that must be resolved.^107^ It is crucial to maintain the functionality of membrane proteins that the cell membrane is effectively oriented and coated on nanomaterials. However, research has revealed that almost 90% of the core NPs are only partially covered by the cell membrane. The extent of coating significantly affects the internalization of these biomimetic nanostructures by cells. Individual cells specifically uptake NPs with a high coating degree (≥50%), whereas those with a low coating degree (<50%) rely on appropriate NP aggregation to enter cancer cells through a cooperative mechanism.^108^ Hence, it is crucial to conduct a comprehensive and careful evaluation of the complete proportion of cell membrane coating to attain optimal tumor targeting.

Additionally, the method of discharging cargo from NPs that are disguised with T cell membranes is yet to be fully understood. The identification of the mechanism of drug release will significantly enhance the development of these nanomaterials. Thus, by integrating molecular simulations with experimental analysis, the endocytic entry mechanism for these nanoplatforms, as well as their drug release mechanism, could be understood in detail.^108,109^ This essential understanding will enhance the logical creation of T cell membrane-covered NPs inspired by nature and open up possibilities for more efficient treatments for cancer.

**Figure 13:**
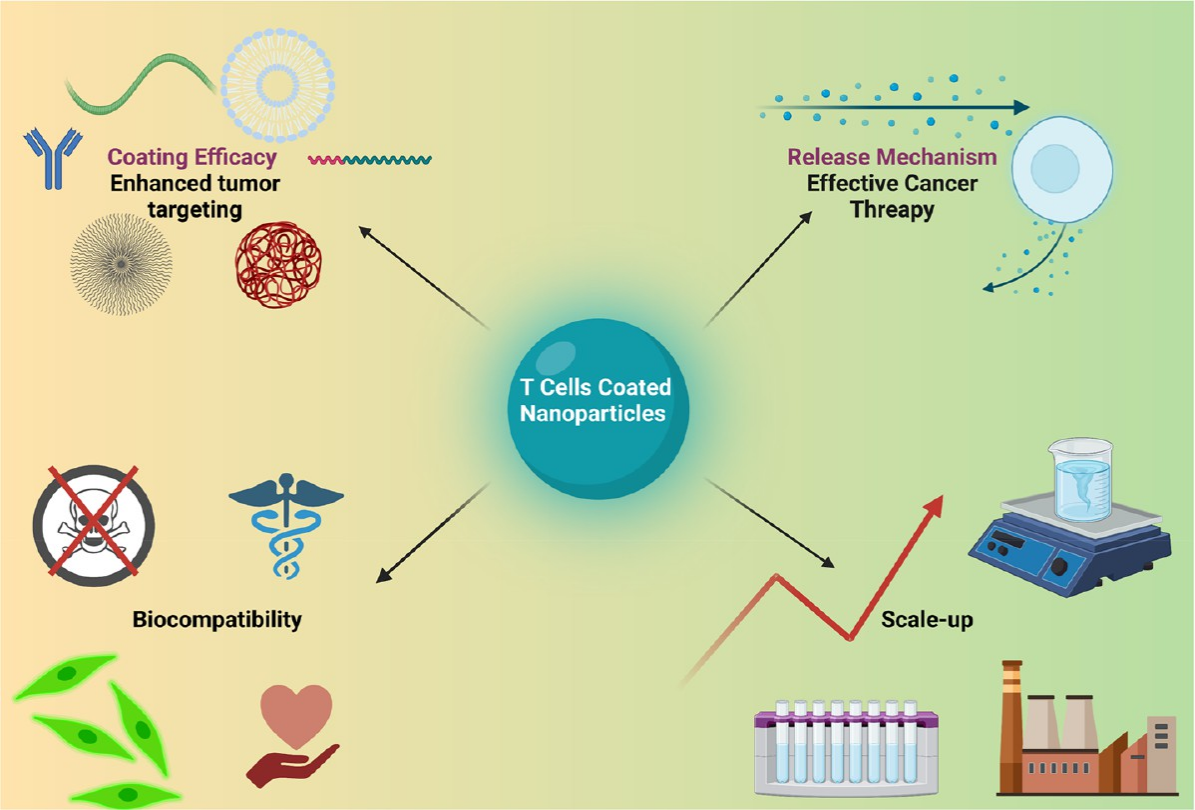
Illustrative demonstration of obstacles in clinical translation of nanoparticles coated with T cells. The authors generated this figure with BioRender (https://www.biorender.com/).

Demonstrating the biocompatibility of NPs coated with T cell and NK cell membranes is an essential prerequisite for advancing to clinical trials. Presently, biomimetic technology utilizing cell membranes demonstrates superior targeting capabilities and biocompatibility in contrast with conventional synthetic delivery systems, which are primarily foreign substances with the potential for immunogenic and toxic reactions (Figure 13). Unlike synthetic coatings, T cell membranes are inherent to the body, thus they possess greater biocompatibility and perform various biological functions akin to their parent cell. Despite the fact that several studies have proved short-term biocompatibility, significant hurdles must be handled before these nanocarriers continue to grow and advance to the clinic.^110^ The accumulation of such substances with a significant fraction in healthy tissues may pose a risk. Besides, modified T cell membranes may potentially raise health risks by inducing hyperinflammatory conditions due to the release of pathological mediators. Therefore, it is crucial to acquire a more profound comprehension and awareness of the interplay among the constituents of the cellular membrane and the organic milieu within the organism.

Another question to be addressed before going to clinical translation is the scale-up of this technology. A simple, reliable, and standardized protocol is necessary to facilitate the industrial production of T cell membrane-coated NPs. The prevalent techniques employed in the production of these NPs are sonication, extrusion, and microfluidics. Currently, the extrusion method is frequently used in laboratories because of its high uniformity, but it has low-yield efficiency, which makes it difficult to scale up for industrial manufacturing. However, the microfluidic method shows promise for mass production of NPs coated with T cell membrane in the future.^109^ Additionally, the purification and characterization procedures for cell membranes vary among laboratories, which leads to uncertainties regarding the physicochemical properties of T cell membranes.^110^ Therefore, it is crucial to publish more scientific information and establish a consistent, highly reproducible methodology for assessing cell membrane integrity.

As of the date of this review, there has been no clinical implementation of T cell membrane-coated nanoplatforms in the realm of cancer therapy. Consequently, this technology is still in its early stages and has not been employed in a medical environment because of apprehensions about its safety, consistency, durability, and variations among individuals.^13^ If the abovementioned limitations are successfully addressed, it would allow such innovative nanocarriers to be employed for the treatment of tumors. We have a positive outlook that in time, the exploration and innovation of nanocarriers camouflaged with T cell membrane will provide immeasurable benefits to the well-being of mankind.

## 8 Conclusion and Outlook

The efficacy of a therapeutic agent is directly related to its capability to selectively target the site of interest (diseased tissue) to defeat the biological hindrance and intelligently release the therapeutic agents in response to the disease environment. The therapeutic effectiveness of chemotherapeutic agents can be magnified, and their toxic effects can be reduced greatly if high concentrations of them could selectively reach to the desired site (tumor tissues) only. Thus, the development of biomimetic nanocarrier systems that provide tumor specific targeting has been increasingly at the vanguard of medical sciences.

Nanoparticles that are coated with cellular membranes have demonstrated to be feasible nanoplatforms with exceptional compatibility with living organisms. Biomimetic nanosystems that are constructed with T cell membranes have been widely utilized to enhance precise administration of medications for treating cancer. These biomimetic nanoparticles have garnered considerable attention because of their unique biological traits. To attain benefits like prolonging blood circulation, evading the immune system, and targeting specific sites, a top-down approach has been devised to create biomimetic nanosystems based on T cell membranes.

The precise targeting capability of biomimetic nanoparticles is heavily reliant on the immune proteins located on cell membranes. Nonetheless, these immunological membranes have a propensity for unfavorable biological impacts that hinder their potential application. Additionally, the immunogenicity linked with major histocompatibility complex molecules on these membranes raises concerns that necessitate additional investigation. Hence, it is essential to shift away from nanoparticle covering to the retrieval of individualized cell membranes for customized uses. The inclusion of nanodrugs in biomimetic T cell membranes can enhance the therapeutic efficacy of nanomedicines by mimicking normal cells. The use of immune cell membranes, particularly, has transformed the domain of targeted drug delivery because of their exceptional compatibility with living tissue and exactness. However, the application of biomimetic nanosystems in tumor therapy that mimic T cells is still a distant possibility in clinics. The complicated and inefficient preparation process of T cell membrane coatings limits their further use. Besides, the specific functional proteins and mechanisms of the structural units on T cell membranes require further confirmation. Additionally, the immunogenicity and potential cytotoxicity of these biomimetic nanosystems need to be thoroughly investigated before their clinical application. The present preparation of these biomimetic nanosystems entails numerous stages that may result in dissimilarities in the procedure. Thus, crucial attributes, like purity, necessitate additional clarification. Likewise, the progress of T cell membrane-enveloping procedures for tumor management is still ongoing. The extensive development of these cell membranes is necessary for the treatment of cancer and other medical applications in the future. Furthermore, apart from cancer treatment, it is needed to devise therapeutic approaches to combat disease-causing agents, such as diverse viruses and bacteria. Research is scarce in these specific domains within the existing body of literature. Research on nanoparticles coated with cell membranes from key immune response cells, such as T cells and NK cells, shows great potential for treating many disorders.

Despite their uniqueness, biomimetic nanoparticles based on T cell membranes are currently undergoing development and face several challenges that must be addressed before they can be translated from the lab to clinical applications. The development of nanoparticles using biomimicry of T cell membranes will continue to be the subject of extensive and meticulous research, with the goal of enhancing the detection and treatment of cancer.
